# Two Adjacent cis-Regulatory Elements Are Required for Ecdysone Response of *Ecdysone Receptor* (*EcR*) *B1* Transcription

**DOI:** 10.1371/journal.pone.0049348

**Published:** 2012-11-14

**Authors:** Hiroyuki Shirai, Manabu Kamimura, Junichi Yamaguchi, Shigeo Imanishi, Tetsuya Kojima, Haruhiko Fujiwara

**Affiliations:** 1 Department of Integrated Biosciences Graduate School of Frontier Sciences, The University of Tokyo, Kashiwa, Chiba, Japan; 2 National Institute of Agrobiological Sciences, Ibaraki, Japan; St. Georges University of London, United Kingdom

## Abstract

Three distinct classes of nuclear receptors, EcR, E75, and HR3, are key regulators in the ecdysone-inducible gene activation cascade in insects. The transcription of these genes is induced by ecdysone (20E) differently, although the detailed mechanisms underlying their responses to 20E are largely unknown. We identified ecdysone response elements (EcREs) present in the promoters of genes coding BmEcR-B1, BmE75-A, and BHR3-B isoforms from *Bombyx mori* employing luciferase reporter assays in an ecdysteroid-responsive cultured cell line, NIAS-Bm-aff3 (aff3). The EcRE of *BmEcR-B1* at −2800 comprises of two adjacent elements separated by 5 bp, E1 (15 bp) and E2 (21 bp), both of which are required for the 20E response. Further analysis using electrophoretic mobility shift assays showed that E1 binds to the EcR/USP heterodimer and that E2 may bind to the E-box (CACGTG) binding factor such as bHLH protein. The unique E1+E2-type EcRE is also detected in the promoter upstream regions of *EcR-B1* from seven lepidopteran species studied. In contrast, both a 20 bp EcRE identified in the promoter of *BmE75-A* and a 18 bp EcRE identified in the *BHR3-B* promoter, contained only E1-type EcR/USP binding element but the E2 type element was not in the promoter regions of these genes. The combination of presence of the E2 element or other cis-regulatory elements in promoter regions explains the different 20E response of each class of nuclear receptor genes. Furthermore, the E1+E2 structure for EcR-B1 can be involved in a possible cross-talk between ecdysteroid and other regulatory pathways.

## Introduction

The steroid hormone ecdysteroid, primarily 20-hydroxyecdysone (20E), coordinates various developmental and physiological processes in insects [1]. The changing titer of ecdysteroid in hemolymph during molting and metamorphosis induces orchestrated gene expression in target tissues. The hormone pulse leads to the direct induction of a few early genes, whose products activate a few early-late genes and inactivate early genes, and further induce hundreds of late genes. The distinctive and sequential expression of each gene is so called the “ecdysone cascade”. Molecular studies have revealed that the early and early-late genes encode transcription factors and that a complex regulatory network coordinates the ecdysone cascade [2]–[4].

Many but not all of the early- and early-late genes are members of the nuclear receptor super-family [3]. Ten nuclear receptors E75, HR3, HR4, EcR, USP, FTZ-F1, HR51, SVP, HR38, and HR39 have been identified as those required for the ecdysteroid signal transduction during metamorphosis [5]. Nuclear receptors, ecdysone receptor (EcR) and ultraspiracle (USP) form heterodimer complex that binds to 20E and regulates the expression of target genes in stage- and tissue-specific manners [4], [6], [7]. The EcR isoforms, generally A and B1 in Lepidoptera, share the same exons encoding the DNA-binding and ligand-binding domains but differ in structure in their N-terminal A/B domain [4], [8]. The alternative expression of EcR isoforms is reported to correlate with the cellular fate of several tissues in holometabolous insects [9]–[12], and thought to be a key step in regulating morphogenesis and tissue differentiation during metamorphosis. However, the control mechanism of EcR isoforms expression is largely unknown at present. Other members of nuclear receptors, E75 and hormone receptor 3 (HR3), a vertebrate RORα homologue in insects, are also known to have several isoforms that differ in structure in the N terminus and to be key regulators in the ecdysone-inducible gene activation cascade [3], [4].

Expression of the three nuclear receptors, EcR, E75, and HR3, are induced by ecdysteroid. *EcR* and *E75*, categorized as early genes, show a rapid transcriptional induction by a low concentration of 20E in the absence of protein synthesis [2], [3]. *EcR*, whose expression is induced earlier by a lower concentration of 20E, is further classified as early class I. *E75*, whose expression is induced later by a relatively higher 20E concentration during wandering stages, is classified further as early class II [2]. By contrast, *HR3* is induced later by a higher concentration of 20E, needs protein synthesis for its full expression, and is categorized as an early-late gene [2], [13]. Thus, 20E induction of transcription of distinct classes of these nuclear receptors genes is different, and these differences may lead to regulation of the stream of ecdysone cascade in the developmental timing. However, the mechanisms responsible for their ecdysone-dependent induction have not been addressed.

It is known that the EcR/USP heterodimer regulates the expression of target genes through binding to *cis*-regulatory DNA sequence called the ecdysone response element (EcRE) in their promoter region. There is currently a sizable list of ecdysone-regulated genes whose EcREs have been characterized. For example, *Hsp27* [14], *Eip28/29* [15], *Fbp1* [16], and *Ddc* [17] are induced promptly by 20E and are known to have the EcRE in their promoter region. However, not much information about the EcRE present in the promoters of ecdeysone-inducible transcription factors in the ecdysone cascade is available.

In this study, we identified EcREs present in the promoters of three ecdysone- inducible nuclear receptor genes using a transient reporter assays in *Bombyx mori* aff3 cells, which are responsive to 20E. We succeeded in showing that the cis-regulatory sequences responsible for 20E-dependent activation are located in an upstream region of *EcR*, *E75*, and *HR3*.

## Results

### Genomic structures of *BmEcR*, *BmE75* and *BHR3*

**Figure 1:**
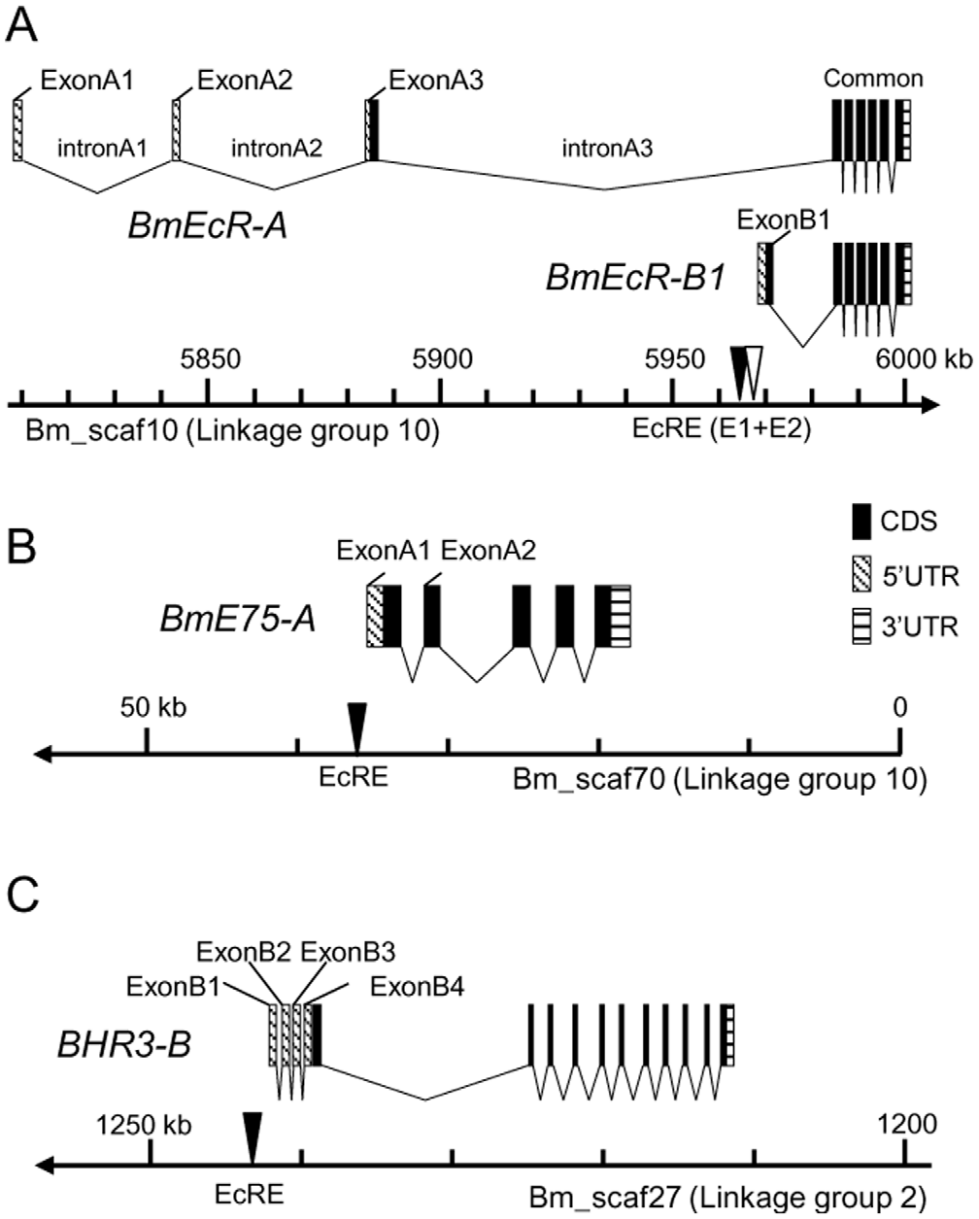
Genomic structure of three nuclear receptor genes in *Bombyx mori*. Exon and intron structures for (A) *BmEcR-A and –B1*, (B) *BmE75-A* and (C) *BHR3-B* are shown on each chromosomal location. The filled box is coding sequence (CDS); shaded box, 5′ untranslated region (UTR); striped box, 3′-UTR. The EcR/USP binding sites (EcRE or E1 for BmEcR-B1) and E2 for *BmEcR-B1* identified in this study are shown by filled and blank triangles, respectively.

Based on the silkworm genome sequence (Scaffold version 2.3), *BmEcR* is mapped on linkage group 10 and its entire region is included in one scaffold, Bm_scaf10. Comparing the sequences with the cDNA sequences of *BmEcR* isoforms, we found that there are three A-specific exons (exons A1 to A3) and one B1-specific exon (exon B1) upstream of the common exons. The transcriptional start sites for A and B1 isoforms are about 160 kb apart from each other (Fig. 1A, Table S1). Similarly, we clarified the genomic structures of *BmE75* and *BHR3*. *BmE75* encodes three isoforms, A to C, and we mapped it to linkage group 10. The exons for *BmE75-A* are included in Bm_scaf70 (Fig. 1B, Table S1). *BHR3* is mapped to linkage group 2 and has two isoforms, of which the B form region is included in Bm_scaf27 (Fig. 1C, Table S1). To screen EcREs, we cloned 5′-flanking genomic fragments from −3652 to +248 for *BmEcR-B1*, from −5032 to +85 for *BmE75-A*, and from −2891 to +18 for *BHR3-B*.

### *BmEcR-B1*, *BmE75-A* and *BHR3-B* respond differently to 20E in aff3 cells

We first examined ecdysone responsiveness of two different *Bombyx* cells, aff3 and BmN (Fig. S1A). The aff3 cell line was aggregated in response to the addition of 0.2 µg/mL and 2 µg/mL 20 hydroxyecdysone (20E) to the cultured medium (Fig. S1A, white arrows). BmN, however, showed no noticeable cellular response to 20E and thus we selected aff3 cells for further experiment. We analyzed the 20E response of endogenous *BmEcR-A*, *-B1*, *BmE75-A*, and *BHR3-B* and found that all of them were induced by 20E clearly in the aff3 cell (Fig. S1B). We also observed that the dose response to 20E differed among *BmEcR-B1*, *BmE75-A*, and *BHR3-B* in aff3 cells. *BmEcR-B1* mRNA was induced by a very low concentration of 20E, 2.0×10^−4^ µg/mL, *BmE75-A* by 2.0×10^−2^ µg/mL, and *BHR3-B* by a high concentration of 20E (2.0 µg/mL) (Fig. S1C).

**Figure 2:**
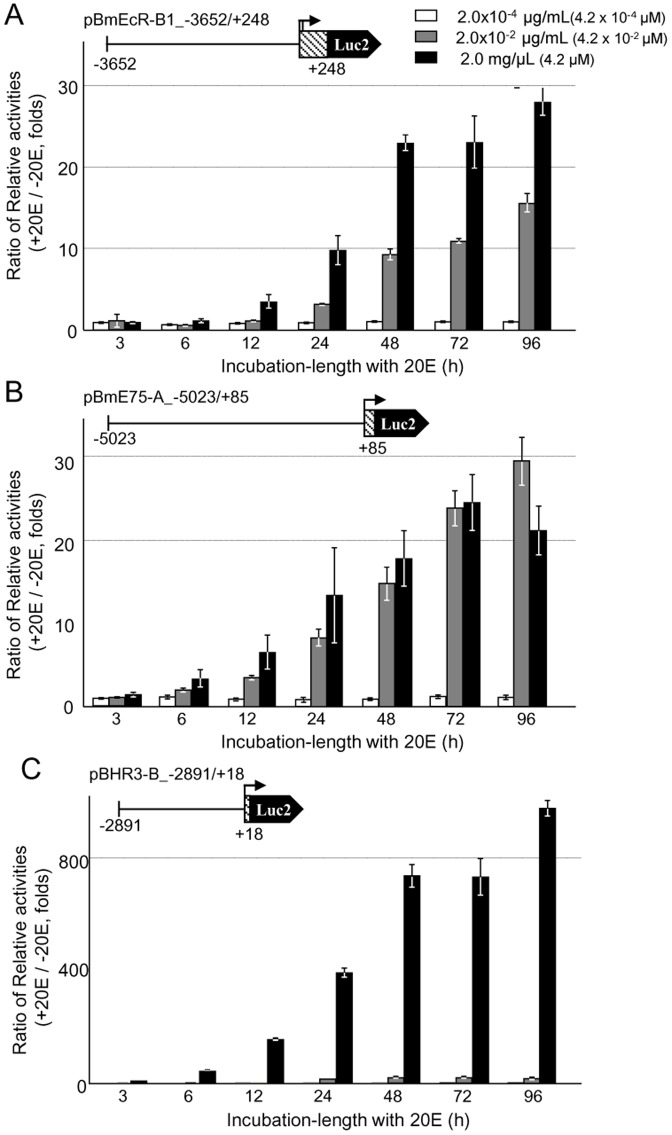
Dose response to 20E of each promoter region for three nuclear receptor genes. Each promoter activity for the full length plasmid of *BmEcR-B1* (A, pBmEcR-B1_−3652/+248), *BmE75-A* (B, pBmE75-A_−5023/+85), and *BHR3-B* (C, pBHR3-B_−2891/+18)) under 2.0×10^−4^(white bar), 2.0×10^−2^ (grey bar) and 2.0 µg/mL (filled bar) of 20E in aff3 cells is shown at each incubation time (h). The ratio of relative luciferase activities with and without 20E (fold induction by 20E) is shown (reference 1.0 at 96 h without 20E). Error bars represent standard error (SE) (N = 4).

Using the luciferase reporter assay, we next tested the 20E response of upstream regions for each gene (Fig. 2). Because the short downstream region of the transcription start site of *BmEcR* is important for its transcription [18], we constructed reporter plasmids that included both upstream and short downstream regions for *BmEcR-B1*, *BmE75-A*, and *BHR3-B*. Seventy-two hours after the transfection of each plasmid, three different concentration of 20E (2.0×10^−4^, 2.0×10^−2^, and 2.0 µg/mL) was added to aff3 cells and the cells were incubated up to 96 h. The average value of the basal activity for the firefly luciferase 2 without 20E in the medium after 96 h culture was 32171 (N = 4) for pBmEcR-B1_−3652/+248, 370221 (N = 4) for pE75-A_−5023/+83, and 30974 (N = 4) for pBHR3B_−2891/+18, respectively. To standardize the luciferase activity, we calculated the relative activities for the firefly luciferase 2 and the *Renilla* luciferase (fluc/rluc). Then, the ratio of the relative activities of luciferase with and without 20E in the medium was calculated and the fold induction by 20E (reference, 1.0 at 96 h without 20E) is shown in figures. Three constructs showed a clear 20E-dependent induction of the luciferase activity: about 30-fold-induction in *BmEcR-B1* (Fig. 2A) and *BmE75-A* (Fig. 2B), and about 1000 fold-induction in *BHR3-B* (Fig. 2C). The results suggest that these constructs include major *cis*-regulatory elements that are involved in the 20E inducible transcription. We did not detect a clear 20E response for BmEcR-A promoter regions (−3932 to +309) (data not shown) and focused on the above three promoter regions for further analyses. Transcription of *BmEcR-B1* and *BmE75-A* constructs was induced at 2.0×10^−2^ µg/mL of 20E and transcription of *BHR3-B* at 2.0 µg/mL of 20E (Fig. 2C). In the following experiments, we analyzed the promoter activities after 48 h of incubation with 20E at 2.0 µg/mL, at which a clear response to 20E was observed in all cases.

### The cis regulatory elements for 20E induction of BmEcR-B1 consist of two adjacent sequences

**Figure 3:**
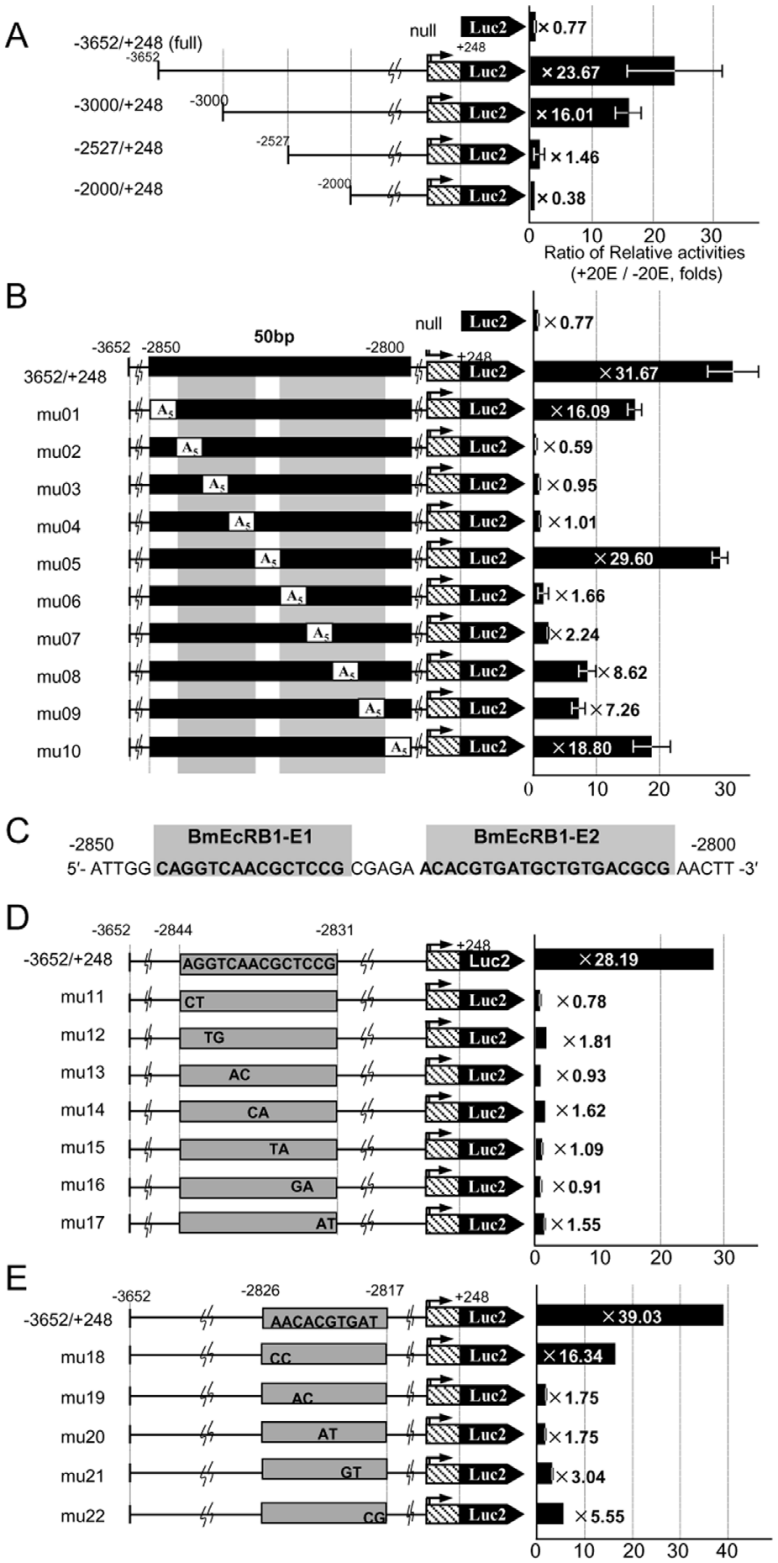
Identification of the ecdysone responsive region in the *BmEcR-B1* promoter. (A, B) Each promoter activity for 5′-deletion series of constructs for the *BmEcR-B1* promoter region (A) and A_5_ mutation series of constructs for BmEcR-B1 −2850 to −2800 (B) is shown, respectively. (C) BmEcRB1-E1 (15 bp, from −2835 to −2830) and BmEcRB1-E2 (20 bp, from −2825 to −2805) were identified as essential for the 20E response elements. (D, E) Each promoter activity for the 2 bp-mutated (A: C and T: G transversion) series of constructs for BmEcRB1-E1 (−2844 to −2831) (D) and for BmEcRB1-E2 (−2826 to −2817) (E) is shown. Each plasmid construct was transfected into aff3 cells and incubated 48 h with 2.0 µg/mL (4.2 µM) of 20E and measured the luciferase activity. The ratio of relative luciferase activities with and without 20E (fold induction by 20E) is shown on the right, as referred 1.0 at 48 h without 20E. Error bar represents SE (N = 4). “null” indicates the pGL4.10 vector.

We next tried to identify the regulatory elements responsible for 20E induction in the construct of *BmEcR-B1* by making a series of deletion mutants. Reporter assays for four deletion mutants from −3651 to −2000 revealed that the region from −3000 to −2527 includes target elements, because the fold induction of the reporter activity by 20E was high in a construct −3000 to +248 (16.01) but drastically reduced in a construct −2527 to +248 (1.46) (Fig. 3A). Similarly, a series of 100 bp-deletions from −3000 to −2527 (Fig. S2A), a series of 40 bp excisions in the region −3000 to −2800 (Fig. S2B), and a series of 10 bp excisions in the region −2850 to −2800 (Fig. S2C) showed that −2850 to −2800 is critical for the 20E response of *BmEcR-B1*.

To clarify further the *cis*-elements for *BmEcR-B1*, we introduced a series of 5 bp-replacements with 5′-AAAAA-3′ (A_5_ mutations) in the region from −2850 to −2800 of pBmEcR-B1_−3652/+248 (Fig. 3B). The luciferase activity decreased significantly in two separated regions, within −2845 to −2830 (mu02 to 04) and within −2825 to −2805 (mu06 to 09). We have named the former 15 bp tract BmEcRB1-element1 (E1) and the latter 20 bp tract BmEcRB1-element2 (E2) (Fig. 3C). Because all 2 bp-mutation series in the BmEcRB1-E1 abolished the 20E-dependent activation (Fig. 3D), the entire 15 bp seems to be essential for the activity. In BmEcRB1-E2, we found that the activity was reduced significantly by the mutations at CACGTG from −2824 to −2819 (Fig. 3E, mu19 to mu21). This palindrome sequence is called class B E-box, which is known to be recognized by several bHLH and bHLH-PAS transcription factors [19]. In summary, the 20E response element in *BmEcR-B1* is located far from its transcriptional start site and comprises two elements, both of which are essential and functionally coupled with each other.

**Figure 4:**
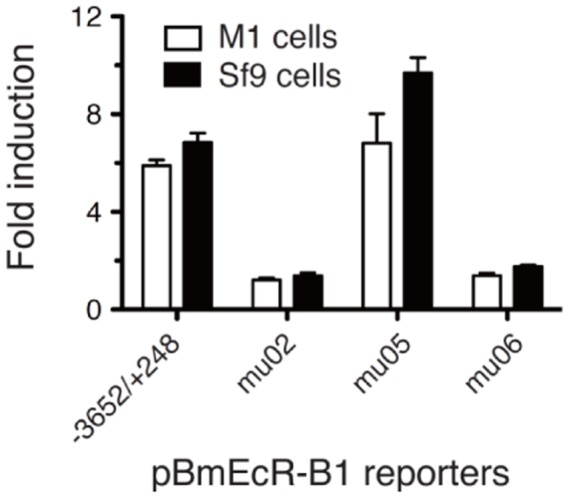
Responses of EcR-B1 reporters to 20E in M1 and Sf9 cells. M1 and Sf9 cells were transfected with representative EcR-B1 reporter plasmids (see Fig. 3), treated with 1 µM of 20E during 3 days, and a dual reporter assay was conducted. −3652/+248, the full length reporter construct; mu02, an E1 region mutant; mu05, a mutant within the internal region between E1 and E2; mu06, an E2 region mutant. Bar represent SE (N = 4).

To know whether E1 and E2 elements for *EcR-B1* are functional in other cell lines, we also performed reporter assays using the silkworm NIAS-Bm-M1(M1) cell that derived from embryos, and the *Spodoptera* Sf9 cell (Fig. 4). A full length reporter construct −3652/+248 and mu05 that is a mutant within the internal region between E1 and E2 (Fig. 3B) showed 20E induction of the reporter activity in M1 and Sf9 cells. However, an E1-region mutant mu02 and an E2-region mutant mu06 did not show an obvious 20E induction of the reporter activity in both cells. This result indicates that E1 and E2 elements play an important role for induction of the *EcR-B1* expression by 20E in multiple cell lines among Lepidoptera.

### The 20E response element for *BmE75-A* and *BHR3-B*

**Figure 5:**
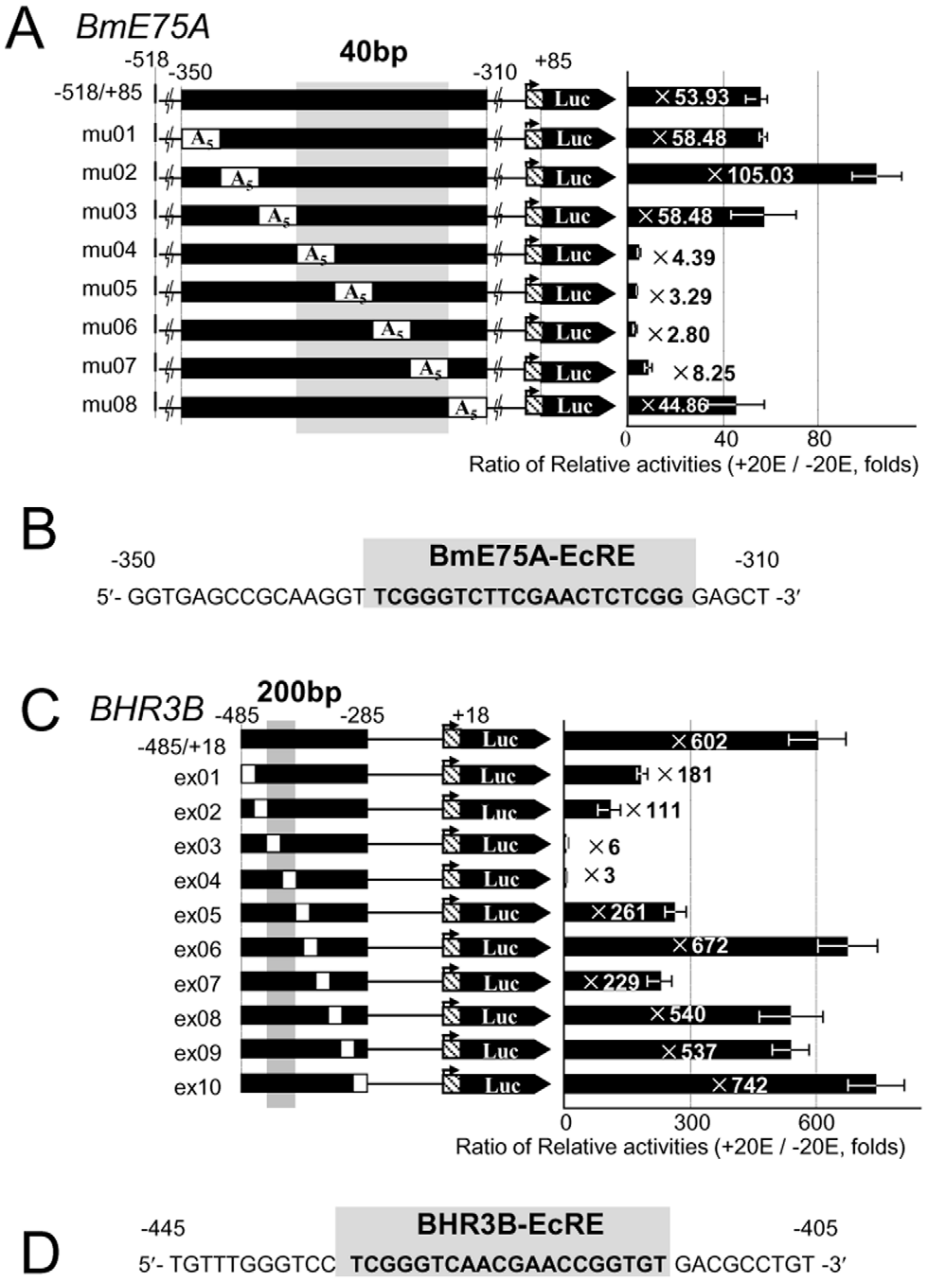
Identification of EcREs for *BmE75-A and BHR3-B*. (A) Each promoter activity of the A_5_ mutation series of constructs for BmE75-A −350 to −310 is shown. (B) The sequence of BmE75A-EcRE (20 bp). (C) Each promoter activity of the excision series of constructs for BHR3-B −485 to −285 is shown. Each excision size (white region) is 20 bp. (D) The sequence of BHR3B-EcRE (20 bp). (A, C) The fold induction by 20E of each construct is shown on the right. Error bars represent SE (N = 4).

Initial screening of the *BmE75-A* promoter region showed that a 20E response element is included in the region from −350 to −308 of *BmE75-A* (Fig. S3A, B). Further analyses of the region from −350 to −310 by A_5_ mutations showed that the constructs from −335 to −315 (Fig. 5A, mu04 to 07) significantly reduced the 20E-dependent activation. We conclude that this 20 bp tract, named BmE75A-EcRE, is essential for the 20E response of *BmE75-A* (Fig. 5B).

Initial screening of the *BHR3-B* promoter region restricted the region from −485 to −285 for the 20E response (Fig. S4), and further 20 bp-excision analyses revealed that the region from −445 to −405 (Fig. 5C, ex03 and ex04) is essential. At the center of this region, we found a 20 bp tract, named BHR3B-EcRE (Fig. 5D), which is very similar to BmE75A-EcRE described above (Fig. 5B). These results indicate that the 20E response element for *BmE75-A* and *BHR3-B* is a simple conserved tract near the transcriptional start site and that this tract differs from the unusual structure of *BmEcR-B1*.

To know the functional role of respective EcREs for an early gene *BmE75-A* and an early-late gene *BHR3-B* on the dose response to 20E, we made two additional constructs in which EcREs were swapped each other (Fig. S5A). Then, we compared the reporter activity of a swapped construct with an original plasmid at various concentration of 20E in the aff3 cell (Fig. S5B). We found that their dose response to 20E was basically same between the original and the swapped construct, both for *BmE75-A* and *BHR3-B*, indicating that BmE75-A EcRE *and* BHR3-B EcRE have the same effect on the dose response to 20E and that some other cis-regulatory elements are involved in their different response to 20E.

### BmEcRB1-E1, BmE75A-EcRE, and BHR3B-EcRE are binding sites of the EcR/USP heterodimer

**Figure 6:**
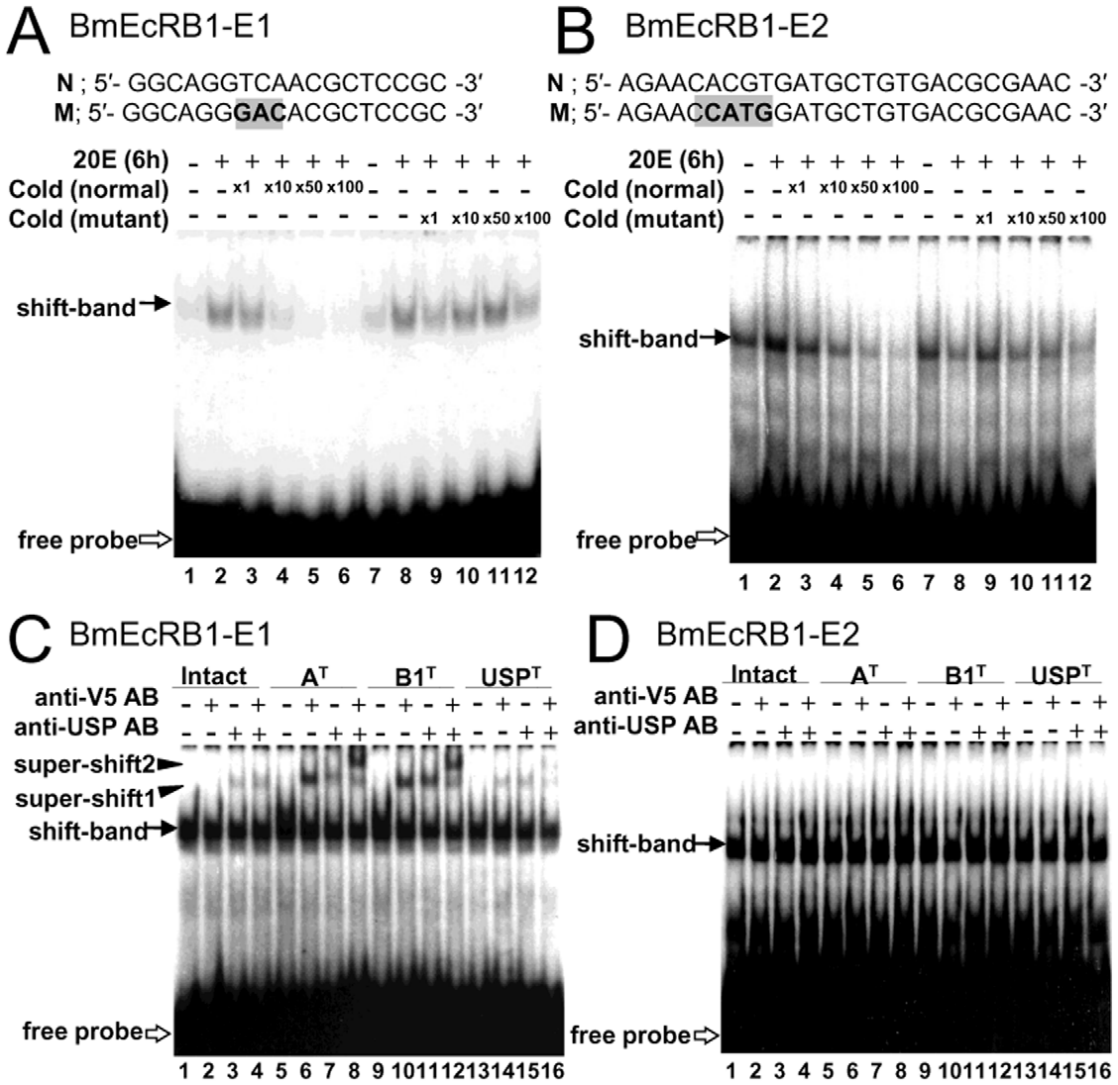
Electrophoretic mobility shift analysis for BmEcRB1-E1 and E2. (A, B) Competition assay with cold probes for BmEcRB1-E1 (A) and for BmEcRB1-E2 (B). Mutation sites in E1 and E2 probe sequence “N” (Normal) are shown in gray region of “M” (Mutant). Two hundred (A) or 50 (B) femtomoles of ^32^P-probe were incubated with 5 µg (A) and 10 µg (B) of cell extracts and loaded onto the gel. 20E (6 h) represents extracts from cells cultured under 20E (2.0 µg/mL). ×1, ×10, ×50 and ×100 represent the ratio of the cold probe amount to the ^32^P-probe amount. Filled arrows show the shifted bands and blank arrows show the free probes. (C, D) Super shift assay with the anti-V5 or/and anti-USP antibody for E1 (C) and for E2 (D). Intact: intact cell extracts. anti-V5 AB: anti-V5 antibody. anti-USP AB: anti-USP antibody. A^T^, B1^T^, and USP^T^ represent extracts from cells that overexpressed EcRA, EcRB1, and USP, respectively.

**Figure 7:**
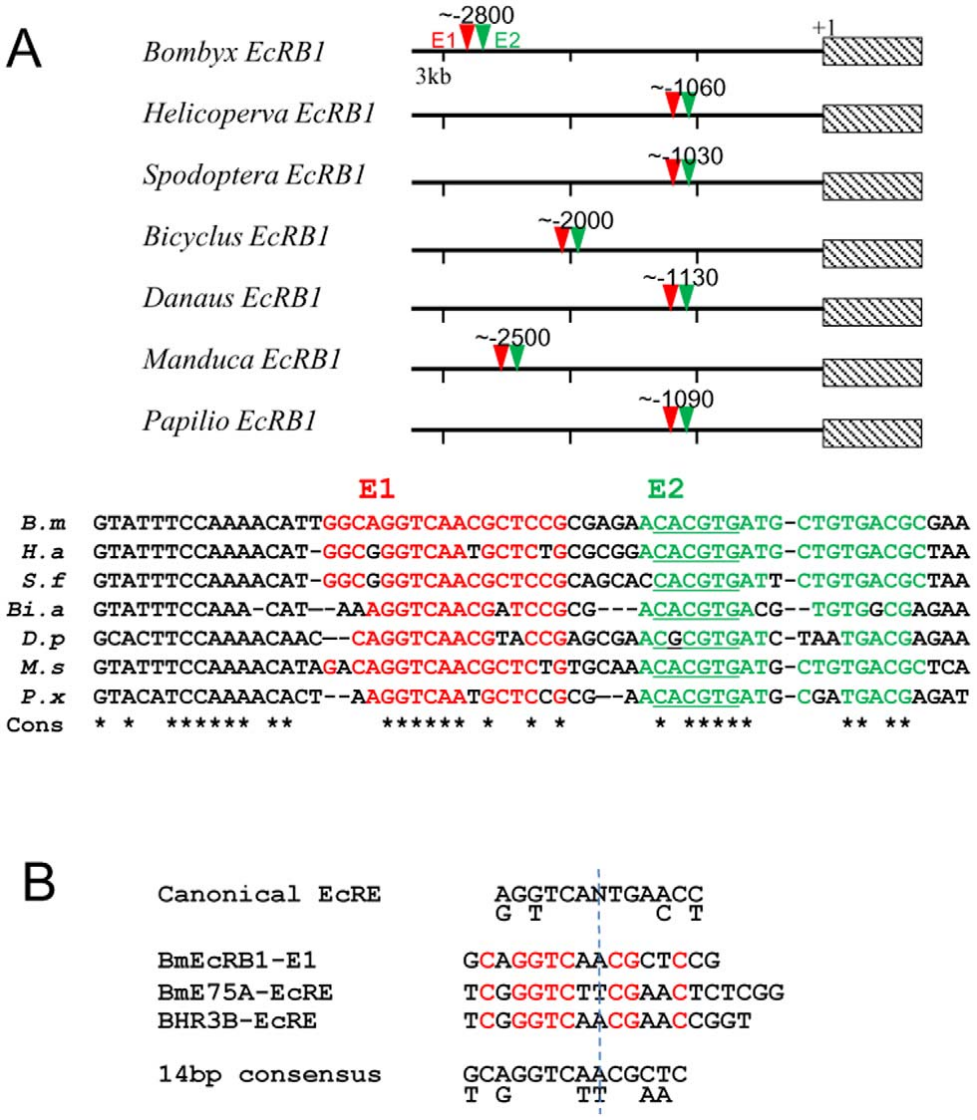
Structural conservation of E1 and E2 structure of EcR-B1 EcRE in Lepidoptera. (A) Schematic localization of E1 (red triangle) and E2 (green triangle) of EcR-B1 EcRE in seven Lepidoptera (upper section). +1, transcriptional start site. Shaded box, EcR-B1 gene. Sequence comparison of each element is shown in lower section. *B. m, Bombyx mori; H.s, Helicoperva armigera; S. f, Spodoptera frugiperda; Bi.a, Bicyclus anynana; D. p, Danaus plexippus; M. s, Manduca sexta; P. x, Papilio xuthus*. E1 (red, nucleotide sequence same to the silkworm E1 element) and E2 (green, nucleotide sequence same to the silkworm E2 element) for lepidopteran *EcR-B1* and their surrounding sequences are aligned. *, consensus nucleotide among seven lepidopteran sequences. E-box in E2 sequence is underlined. (B) The alignment of the EcR/USP heterodimer binding site for ecsysone-inducible nuclear receptor genes, *EcR-B1, E75-A and BHR3-B*. Classical EcRE of *Drosophila* and 14 bp consensus motif among three nuclear receptors found in this study are show in the top and bottom, respectively. The red character shows conserved nucleotide in three elements.

To test whether the *cis*-elements we found are the binding target of the EcR/USP heterodimer, we conducted an electrophoretic mobility shift assay (EMSA) using BmEcRB1-E1 (Fig. 6A and C), BmEcRB1-E2 (Fig. 6B and D), BmE75A-EcRE (Fig. S6A and C) and BHR3B-EcRE (Fig. S6B and D) probes. Each probe was incubated with whole-cell extracts from aff3 cells cultured with or without 20E. BmEcRB1-E1 (Fig. 6A, N), BmE75A-EcRE (Fig. S6A, Normal), and BHR3B-EcRE (Fig. S6C, Normal) proves showed one shifted band whose intensity was increased by the incubation with 20E (compare lanes 1 and 2 in each figure). These three elements have sequence similarity, especially in their 5′-half side (Fig. 7B), and similarity to the consensus sequence of the EcR/USP heterodimer binding site suggested by Cherbas *et al.* [15]. It is reported that USP recognizes 5′-TC(A/T)-3′ in the sequence, and thus we made mutated probes by replacing 5′-TC(A/T)-3′ sites with 5′-GA(C/G)-3′ in three elements [20] (Fig. 6A, Fig. S6A, and Fig. S6B; gray area in M or “Mutant”). Addition of excess amounts of non-labeled BmEcRB1-E1 (Fig. 6A), BmE75A-EcRE (Fig. S6A), and BHR3B-EcRE (Fig. S6B) without mutations to the mixture decreased the intensity of the shifted band (see lanes 3 to 6 in each figure), but cold probes with mutations did not change the intensity of the band (lanes 9 to 12), suggesting that USP binds to these elements in a sequence-specific manner.

To characterize further the binding of the EcR/USP heterodimer to BmEcRB1-E1, BmE75A-EcRE, and BHR3B-EcRE, we overexpressed BmEcR-A (A^T^), BmEcR-B1 (B1^T^) and BmUSP (USP^T^), all of which were tagged with a V5 antigen, in aff3 cells by plasmid transfection. The cell extracts from BmEcR-A-overexpressed (A^T^) and BmEcR-B1-overexpressed cells (B1^T^) intensified shifted bands for BmEcRB1-E1, BmE75A-EcRE, and BHR3B-EcRE (Fig. 6C, Fig. S6C, and S6D; lanes 5 and 9), compared to intact cell extracts (lane 1). In addition, supershifted bands were observed with anti-V5 antibodies in the overexpressed A^T^, B1^T^, and USP^T^ cell extracts (super-shift1; lanes 6, 10 and 14 in each figure), respectively, but not in the intact cell extracts (lane 2). This result suggests that EcRA, EcRB1, and USP are involved in the protein–DNA complex corresponding to the supershifted band and that BmEcRB1-E1, BmE75A-EcRE, and BHR3B-EcRE are the binding sites of the EcR or USP protein. This possibility is also supported by the results with anti-USP antibodies (lanes 3, 7, 11 and 15 in each figure), and by the results of the addition of two antibodies simultaneously, showing that the bands were shifted further (super-shift2; lanes 8, 12 and 16 in each figure), suggesting that the EcR-A/USP or EcR-B1/USP heterodimer binds to each element.

### BmEcRB1-E2 binds to a cellular factor other than the EcR/USP complex

BmEcRB1-E2 mixed with the cell extracts including overexpressed EcRA, EcRB1 or USP did not show supershifting with anti-V5 or anti-USP antibodies (Fig. 6D), suggesting that BmEcRB1-E2 interacts with unknown trans-factors other than the EcR/USP heterodimer. BmEcRB1-E2 contains a binding motif of several bHLH proteins, E-box. Thus, we next tested whether the CACGTG E-box palindrome motif, that was shown to be important for the ecdysone response (Fig. 3E), is truly involved in the binding of some cellular factors. To examine whether this motif is actually bound by trans-factors in aff3 cells, we performed a competition assay using excess amounts of cold probes with or without a 4 bp-mutation (5′-CCATGG-3′) (Fig. 6B, M gray region). Addition of the normal cold probe decreased the intensity of the shifted band (Fig. 6B, lanes 3 to 6), whereas the mutant probe did not (lanes 9 to 12), suggesting that some E-box binding factor binds to BmEcRB1-E2 in a sequence-specific manner.

### The two-component structure of E1 and E2 for *EcR-B1* is conserved among Lepidoptera

When searching the nucleotide database available at present, we found upstream genome fragments for *EcR-B1* in seven lepidopteran species distantly related each other (Fig. 7A). We found that all have highly conserved E1 and E2 sequences with a 2–5 bp interval, CACGTG (E-box) in E2 and a 15 bp conserved sequence upstream of E1, although their genomic locations vary from −2800 in *Bombyx* to −1030 in *Spodoptera*. We could not find a highly conserved sequence in the upstream regions of *EcR* other than the above E1+E2 structure. This observation suggests that the E1+E2 structure is involved in a common regulation system for EcR-B1 in Lepidoptera.

### Highly conservation of EcREs for *HR3-B* and *E75-A* among Lepidoptera

Previous analysis of the promoter region of *Manduca sexta MHR3* suggested four cis-elements in its 20E-dependent transcriptional regulation [13], [21]. We found that BHR3B-EcRE corresponds to MHR3-EcRE1 based on their sequence similarity (17/18 bp identity) and that the BHR3B promoter region has no homologous sequences to other EcREs of MHR3 (Fig. S7A). Furthermore, we found three highly conserved regions between *BHR3B* and *MHR3* (*A–C* blue lines in Fig. S7A). Region B includes BHR3B-EcRE and MHR3 EcRE1 (red boxes), region A includes a BHR3B-EcRE-like sequence (yellow letters) that is homologous to the monomeric response element 1 (MRE1) reported in *Manduca* HR3 (underlined). When the promoter regions for *HR3-B* were compared among four Lepidoptera, we found that BHR3B-EcRE identified here and its upstream regions are highly conserved among all species (Fig. S7A). Similarly, we found that BmE75A-EcRE and its upstream regions are also highly conserved among four lepidopteran species (Fig. S7B).

### Genome-wide screening of conserved 20E response elements in the silkworm genome

**Figure 8:**
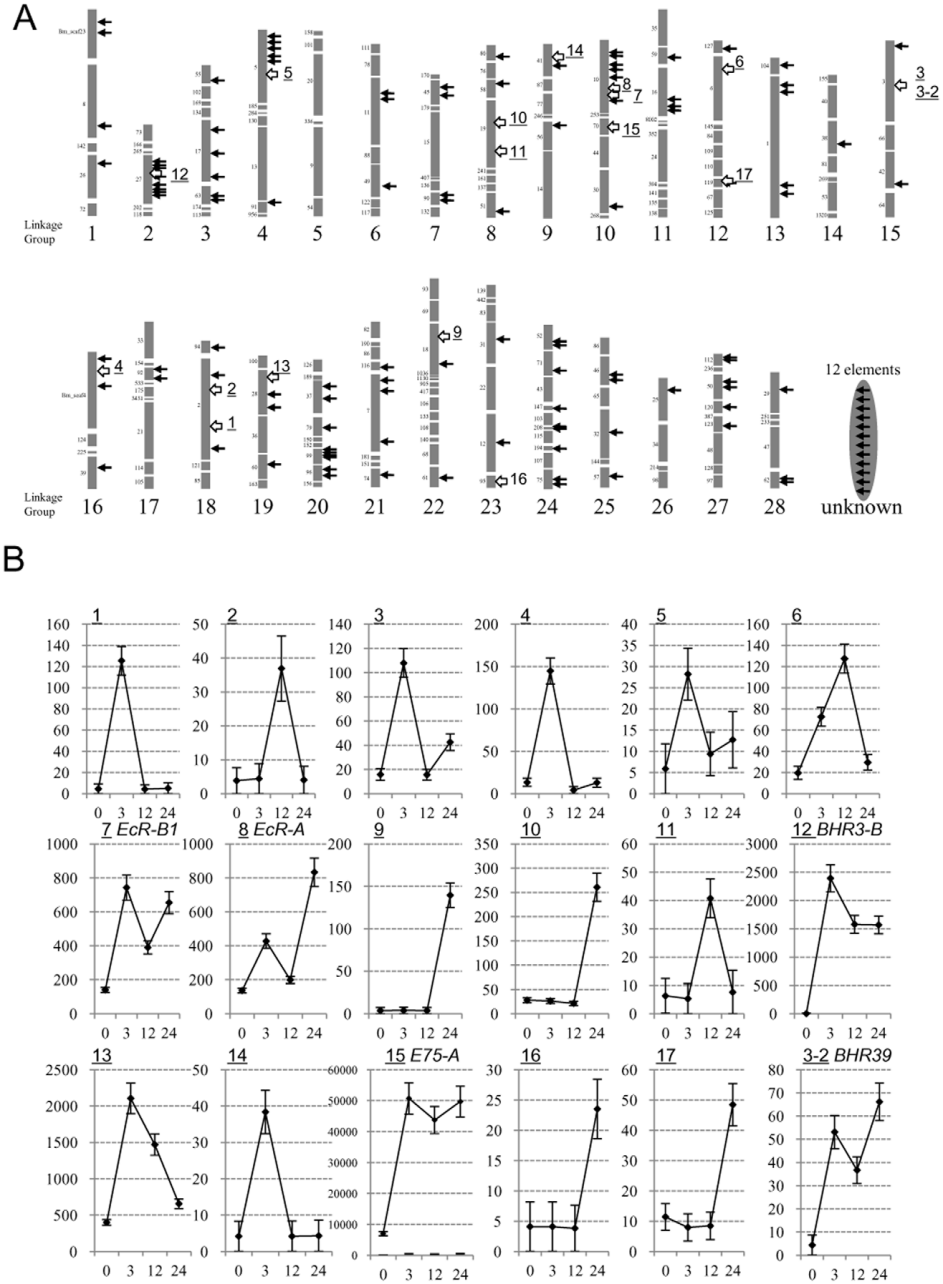
The genome-wide distribution of 14 bp EcRE consensus motif in the silkworm. (A) The location of the genomic sequences identical to 64 patterns of 14 bp consensus motif (5′-KCRGGTCWWCGMWC-3′, see Fig. 7B). The arrows show the location of 138 motifs in each linkage group of the silkworm. The chromosomal locations of 12 motifs are not certified. Each grey bar shows the scaffold composed of each chromosome. The number located left side of grey bars represents the scaffold number. The white regions show the gap between scaffolds. Underlined numbers on the right of grey bars represent the location for 20E activated genes (more than 4 fold induction by 20E) identified by microarray analysis. 3 and 3-2 are located near the same 14 bp consensus motif. (B) The 20E-dependent activation of predicted-genes surrounding 14 bp consensus motifs analyzed by microarray. The relative expression patterns of 18 genes in aff3 cells treated with 20E for 0, 3, 12, 24 h are shown, based on the spot intensities for the predicted genes in the silkworm 44K cDNA microarray. Detailed information for 18 genes and 17 motifs is summarized in Table S6.

We compared BmEcRB1-E1 (16 bp), BmE75A-EcRE (20 bp), BHR3B-EcRE (18 bp) with the canonical EcRE (15 bp), a consensus sequence among dozens of *Drosophila* ecdysone-inducible genes (RGKTCANTGAMCY) (Fig. 7B). The USP-binding half is conserved, but the EcR-binding half varies in sequence and length. These three EcREs for *Bombyx* nuclear receptor genes show the highly conserved 14 bp tract in their 5′-side. The 14 bp consensus motif (5′-KCRGGTCWWCGMWC-3′) among *Bombyx* four nuclear receptor genes has 64 ( = 2^6^) patterns of the sequences (Fig. 7B). We then searched 64 patterns of the sequences in the silkworm genome using a scaffold sequence search program which resides in the KAIKObase (http://sgp.dna.affrc.go.jp/KAIKObase/) and found 138 motifs which matched 100% to either of 64 sequences. Fig. 8A shows locations the motifs in the silkworm chromosomes. Near the 138 motifs, we found 296 surrounding genes.

To know whether these predicted motifs work as ecdysone response elements, we performed a microarray analysis using the silkworm DNA tip tilled with 44000 oligonucleotides. We prepared RNAs from aff3 cells treated with 2 µg/mL 20E during 0, 3, 12, and 24 h, and compared gene expressions at each time by microarray (Fig. 8B), which showed that *BmE75-A*, *BHR3-B*, *BmEcR-B1 and BmEcR-A* were induced by the 20E treatment clearly. Among 138 motifs, there are at least 17 motifs near which gene expressions showed more than 4 fold induction by 20E (Fig. 8B, Table S6). Interestingly, the list includes *Wnt1*, another ecdysone-inducible nuclear receptor gene *BHR39*. These results suggest that the genome-wide screening of the motif is effective to find novel ecdysone inducible genes and many consensus motifs found here may work as ecdysone response elements.

## Discussion

We have here identified essential EcREs in three different ecdysone-inducible nuclear receptor genes, *Bombyx EcR-B1, E75-A, and HR3-B.* Our data clarify EcREs for the upstream transcription factor genes in the ecdysone cascade in Lepidoptera. A dozen EcREs have been reported in many genes from *Drosophila* [14]–[17], [21]–[25], but rarely in upstream genes of the ecdysone cascade. Some groups reported EcREs for the early ecdysone inducible loci to date [26]–[28]. The consensus motif among dozens of EcREs reported to date, canonical EcREs, shows considerable variations, and thus it is difficult to find the critical EcRE candidate based on the sequence similarity on the genome sequence. Our approach by reporter assay, which is first screening 3–5 kb upstream region and then restricting the region including EcRE, was successful to find each critical EcRE element for nuclear receptor genes. From the genomic sequence, it was unexpected that the unusual E1+E2 structure for BmEcRB1 EcRE resides 2.8 kb far upstream of the transcriptional start site, and that BmEcRB1-E1, BmE75A-EcRE and BHR3B-EcRE have a similarity each other and to canonical EcRE (Fig. 7B). We have shown here by EMSA analyses that BmEcRB1-E1, BmE75A-EcRE and BHR3B-EcRE work as the binding site of EcR/USP heterodimer (Figs. 6 and S6). The 5′-half of these EcREs are highly conserved, suggesting that USP binding sites are structurally rigid (Fig. 7B). In contrast, the 3′-half structure of their EcREs are relatively various especially at 3′-terminal regions, which may affect the binding features of EcR molecule to them. An important question is why the ecdysone inducible genes, such as early, early-late and late genes, show different expression profiles. One possible answer to this is that respective EcREs for each class of ecdysone-inducible genes may have different affinity to EcR/USP heterodimer. For example, if EcREs for early class gene have higher affinity to EcR/USP, it enables the rapid induction of the early gene expression by a lower 20E titer. However, the EcRE swapping experiment between *BmE75-A* (early class) and *BHR3-B* (early-late class) denied this possibility, because BmE75A-EcRE and BHR3B-EcRE showed the same effect on the dose response to 20E (Fig. S5). This result indicates that some trans-factors which interact with other cis-regulatory elements in the promoter region of *BmE75-A* and *BHR3-B* enable different expression patterns to 20E dose between early class *BmE75-A* and early-late class *BHR3-B*.

We found that a 14 bp consensus motif (5′- KCRGGTCWWCGMWC-3′) conserved among EcREs for three nuclear receptor genes exists at 138 locations in the silkworm genome and 296 genes surround the motifs (Fig. 8A). Since 135 of 296 genes identified on the silkworm genome are not included in a 44K expression microarray of the silkworm, we have tested 20E-induced expression of the rest 161 genes at 3, 12, 24 h incubation in aff3 cells by the microarray analysis and observed that 18 genes showed more than 4 fold induction of gene expression by 20E (Fig. 8B). In addition to four nuclear receptor genes characterized in this study, *BHR39*, a homologue of *DHR39* [29], showed the 20E induction by the microarray analysis, suggesting that this motif or some similar motifs are used in the 20E response of many nuclear receptor genes of *B. mori* [30]. Because a considerable number of genes are not tilled on the array, and not characterized on the silkworm genome, further studies are necessary to clarify this possibility. Genomic tilling array analyses in *Drosophila* suggested that multiple genomic regions interact with the EcR/USP heterodimer [28]. We also searched the 14 bp consensus motif identified here in *Drosophila* and non-lepidopteran insects, but failed to find similar motif sequences, implying that the EcR/USP-binding sequences became diversified in different orders of insects.

The most interesting finding of this paper is that the EcRE of *BmEcR-B1* is composed of two adjacent elements, E1 (15 bp) and E2 (21 bp), both of which are required for the 20E response. This unique structure of *EcR-B1* EcRE is also conserved in other Lepidoptera, *Helicoverpa*, *Spodoptera*. *Bicyclus*, *Danaus*, *Manduca*, and *Papilio* (Fig. 7A). In addition to the highly conserved E1+E2 structure, the fact that both E1 and E2 sequences are necessary for the 20E induction of the reporter activity in M1 and *Spodoptera* Sf9 cells (Fig. 4), indicates a functional importance of this structure on the EcR-B1 expression among Lepidoptera. Interestingly, the 60 bp sequences surrounding these EcREs are relatively conserved among 7 lepidopteran species (Fig. 7A), but other promoter regions except near the transcriptional start sites are not conserved.

We also searched the E1+E2 sequence in the silkworm genome and found a similar structure in the second intron A2 of *BmEcR-A*, about 100 kb upstream of the *BmEcR-B1*, whereas there are more sequence variations than the conserved E1+E2 for *BmEcR-B1*. The putative BmEcRA-E2 appears to include a CRE-like TGATGTCA instead of CACGTG (class B E-box) of BmEcRB1-E2, suggesting different binding factors between A and B1 isoforms of EcR. We found that the putative BmEcRA-EcRE binds to the EcR/USP heterodimer (data not shown), while its contribution to the 20E response has not been confirmed. We also searched E2-like sequences in other genomes, but we did not find a similar sequence in EcR-B1 promoter regions of *Drosophila* and other non-lepidopteran insects.

Because the introduction of a mutation into the E-box of BmEcRB1-E2 decreased the intensity of the mobility shifted band (Fig. 6B), we speculate that some bHLH protein recognizing the E-box is expressed in aff3 cells. In *Drosophila*, it is reported that 48 transcriptional factors belong to bHLH protein and 7 transcriptional factors belong to bHLH-PAS protein [31]. In addition, 52 and 54 bHLH genes have been identified in *Bombyx mori* and the red flower beetle, *Tribolium casteneum*, respectively [32]–[34], and thus there seem to be many candidates binding the E2 element. Recent studies reported that a Methoprene-tolerant (Met) protein, one of the bHLH-PAS proteins, can bind both EcR and USP [35] and that Met and another bHLH-PAS protein, a steroid receptor coactivator Taiman and FISC, a coactivator of EcR/USP complex mediate the juvenile hormone (JH) -signaling pathway [36]–[38]. Thus, it is of interest to know whether EcRB1-E2 is involved in the cross-talk between the ecdysone and juvenile hormone-signaling pathway. A bHLH-PAS protein Taiman, a coactivator of the EcR/USP complex [39], is another possible candidate for the BmEcR-E2 binding factor. Recently, we have tested a preliminary EMSA with the E2 probe using the cell extracts which include over-expressed His-tagged Met proteins, but failed to detect the noticeable binding of Met proteins to the E2 sequence of BmEcRB1 EcRE (data not shown). We speculate that Met proteins do not bind to E2 sequence, or that some other factors which interact with Met proteins may be necessary for stable binding to the E2 sequence, although further detailed analysis will be needed.

Our former observation that the expression of *BmEcR-B1* but not *BmEcR-A* is repressed by JH [40] support the above notion. To identify whether the unique E1+E2 structure of *BmEcR-B1* is involved in the ecdysone- dependent developmental event, we performed EMSA with the E2 probe in cellular extracts from pupal wings, and observed the shifted band only in the proximal region extract but not in the distal region extract (data not shown). We previously observed that *BmEcR-B1* is expressed in the proximal region but *BmEcR-A* in the distal region of pupal wings [11], [41]. The above result indicates that some BmEcRB1-E2 binding factor is present in the proximal region but absent in the distal region, which may explain how the region-specific expression of *EcR-B1* is involved in the lepidopteran wing formation. We have recently observed that the *Bombyx* Met2 gene is transcribed more dominantly in the proximal region than in the distal region of pupal wings, supporting the above idea. In *Drosophila* glue protein genes, *Sgs-3* and *Sgs-4*, the binding site of a forkhead transcription factor is located near the EcR/USP binding site in their 5′-flanking region and is essential for their ecdysone-dependent induction [23], [24]. Similar to *Drosophila Sgs-3* and *Sgs-4*, the coupling of EcR/USP binding to E1 and a transcription factor binding to E2 may regulate the ecdysone-dependent and tissue-specific expression of *BmEcR-B1* in the silkworm pupal wings.

We compared the BHR3-B-EcRE identified here with putative four EcREs (1 to 4) of *Manduca sexta MHR3* [26], and found that BHR3-B-EcRE corresponds to MHR3-EcRE1 based on the sequence similarity. The MHR3-EcRE1 was shown to interact with EcR-B1/USP1 in *Manduca* GV cells, but the reporter construct composed of the short promoter region including the EcRE1 did not show the 20E-dependent activation of MHR3 [26]. In the upstream region of BHR3-B, however, there are no homologous sequences to EcREs 2, 3 and 4 of MHR3, and thus we speculate that the most intrinsic response element to 20E is MHR3-EcRE1. In *Manduca sexta* GV1 cells, MHR3 expression is suppressed by overexpression of MsE75A, which binds monomeric response element 2 (MRE2) 2.4 kb upstream of *MHR3* at a lower concentration of 20E [13]. We also found an MRE2-like sequence in the upstream region of the BHR3B promoter (5′-TATTAAGGCGATGATAC-3′, −2415 to −2399), suggesting that the HR3 regulation system is conserved between the two species and this type of interaction may underlie the difference in the 20E response between E75 and HR3. Similar to EcR-B1 promoter regions (Fig. 7A), the EcREs in promoters of BHR3-B and BmE75-A and its surrounding regions were highly conserved among four lepidopteran species (Fig. S7), but we could not find similar sequences in HR3 and E75 promoters in other order of insects at present. This suggests that the cis-regulatory sequences for 20E response of ecdysone inducible nuclear receptor genes are diversified among various insect groups and further detailed studies in various insects will depict more general structural features of them.

### Conclusion

The EcRE of *BmEcR-B1* comprises of two adjacent elements E1 and E2, both of which are required for the 20E response, but *BmE75-A* and *BHR3-B* EcREs contained only an E1-type EcR/USP binding element. This indicates that the combination of presence of the E2 element or other cis-regulatory elements in promoter regions explains the different 20E response of each class of nuclear receptor genes. E2 of BmEcR-B1 EcRE includes E-box (CACGTG) and may bind to E-box binding factors such as bHLH protein. Furthermore, the unique E1+E2-type EcRE is highly conserved among the promoter upstream regions of *EcR-B1* from seven lepidopteran species studied. These observations suggest that the E1+E2 structure for EcR-B1 can be involved in a possible cross-talk between ecdysteroid and other regulatory pathways.

## Materials and Methods

### Cell culture

The NIAS-Bm-aff3 (aff3) cell line [42] that derived from female fat bodies of 5th instar larva, NIAS-Bm-M1(M1) cell line [43] that derived from male embryos of the silkworm and Sf9 cells (purchased from Invitrogen) that derived from a Noctuidae moth *Spodoptera frugiperda* were maintained at 27°C in an IPL-41 insect medium (Gibco) with 10% fetal bovine serum (FBS, Gibco). BmN that derived from the silkworm ovary was maintained at 27°C in a Grace's Medium Supplemented (Gibco) with 10% FBS.

### Dual-luciferase assay

The plasmid constructs were transfected into aff3 cells by a liposome-mediated method using the TransFast™ or FuGENE®-HD reagent (Promega). Forty microliters of Sf-900II SFM (Gibco) containing 100 ng of each pGL4.10 construct, the HSP70-pRL plasmid and 1.0 µL of TransFast™ were added to 5×10^4^ cells. The activities of firefly and *Renilla* luciferase in cell lysates were measured by a dual-luciferase reporter assay system (Promega) using a MicroLumat Plus (Berthold). The firefly luciferase activity was normalized to the *Renilla* luciferase activity.

### Electorphoretic mobility shift Assay (EMSA)

The oligonucleotides used as probes are shown in Table S5. We added 10 µg of cell extract into a gel shift buffer (12 mM HEPES [pH 7.9], 60 mM KCl, 65 mM NaCl, 7.5 mM MgCl_2_, 6.6 mM EDTA, 1.2 mM DTT, and 12% glycerol), then 500 fmol of the labeled probe (or with competitor DNA), and the mixture was incubated for 20 min on ice. The anti-V5 antibody or/with anti-USP antibody was added, and the mixture was incubated on ice for another 1.5 h and then separated on a 4% polyacrylamide gel containing 2.5% glycerol. The dried gel was analyzed with a high-performance autoradiography film (GE Healthcare). To produce the anti-USP antibody, a BmUSP (Table S1) peptide [H_2_N-MLDGFRDDSTPPPPFKNYC -COOH] (from 85 to 105 amino acids in A/B region) was synthesized, HPLC purified, and used to immunize rabbits (Sawady Technology (QIAGEN)).

### Plasmid construction

We used the pGL4.10 vector containing a firefly luciferase2 gene as the reporter, for the promoter assay. To normalize the expression of the firefly luciferase2 in aff3 cells, the pRL vector containing the *Drosophila* HSP70 promoter, which constitutively expresses the *Renilla* luciferase in aff3 cells (HSP70–pRL), was co-transfected with each experimental DNA into the firefly luciferase vector.

The deletion and substitution mutants were constructed by inverse PCR and self-ligation with 5′-phosphorylated primers. Phosphorylated primers were made with T_4_ polynucleotide kinase (TOYOBO) and ATP (Roche). The pBmEcR-B1_−3652/+248 plasmid was used as a basal template for making various mutants of *BmEcR-B1*. Similarly, the pBmE75-A_−518/+85 and pBHR3-B_−485/+18 were used as basal templates to make various mutants for *BmE75-A* and *BHR3-B* mutants, respectively. To introduce various mutations, one primer with excisions or A_5_ or 2 bp substitutions were used in the inverse PCR. Each inverse PCR product was amplified by iProof (Bio-Rad) with a pair of primers (Tables S2, S3, S4, S5). After the inverse PCR, the products with the phosphorylated 5′ ends were self-ligated with the TaKaRa DNA ligation kit version 2 (TaKaRa) for 16 h. After isolating the mutant plasmid, the mutated inserts were subcloned between *Eco*RV (BmEcR-B1 mutant) of a pBmEcR-B1_−3652/+248 vector. The mutated inserts of BmE75-A and BHR3-B were subcloned between *Sma*I and *Nco*I of each pBmE75-A_−518/+85 or pBHR3-B_−485/+18.

The BmEcR-B1_ORF and BmUSP_ORF were amplified by PCR with iProof (Bio-Rad) using primer sets designed to make sequences for *Eco*RI or *Not*I and at each end of the PCR product (Tables S2, S3, S4, S5). The PCR products digested with *Eco*RI and *Not*I were cloned into the piZT/V5-His vector (Invitrogen). We named these constructs B1^T^ and USP^T^. BmEcR-A_ORF was cloned into the piZT/V5-His vector, and then partially exchanged of B1^T^ C-terminal by *Eco*RV and *Not*I. This construct was named A^T^.

### Cell extract preparation

At 120 h after transfection of overexpression vectors of BmEcR-A, BmEcR-B1 and BmUSP, cell pellets were lysed in 200 µl of a sonication buffer (60 mM KCl, 25 mM HEPES [pH 7.5], 10% glycerol, 1 mM EDTA, 1 mM DTT, 0.5 mM PMSF, 1 mg of antipain per ml), sonicated at 4°C for 35 s, and centrifuged at 12,000×g at 4°C for 10 min. The supernatant was collected as 20 µl aliquots and stored at −70°C.

### Northern hybridization

Approximately 20 µg total RNA extracted from aff3 cells under various 20E conditions was separated on a 1.0% denaturing gel (18% formaldehyde, 20 mM MOPS (3-morpholinopropanesulfonic acid, pH 7.0), 5 mM sodium acetate, 1 mM EDTA, and 1.0% agarose), and blotted onto Biodyne A nylon membrane (Pall BioSupport) in 10×SSC (1×SSC is 150 mM NaCl, 15 mM sodium citrate). The membranes were hybridized with each probe at 42°C for 16 h in 50% formamide, 10×Denhardt's solution (0.2% each of BSA, Ficoll, and polyvinylpyrrolidone), 50 mM sodium phosphate (pH 7.0), and 2.5 µg/ml denatured salmon sperm DNA in 5×SSC. Post-hybridization washes were carried out once each in 2×SSC with 0.1% SDS for 15 min at 42°C. PCR primers used are referred to Tables S2, S3, S4, S5.

### Microarray analysis

A silkworm 4×44K oligonucleotide microarray (order made by Agilent) is tilled by 44,000 olidonucleotide probes (60-mer) for the silkworm genes. Each slide has four arrays. After the aff3 cells were incubated with 2.0 µg/mL of 20E for 3, 6, 12 and 24 h, total RNAs were extracted at each time. The concentration of total RNA was checked by a NanoDrop ND-1000 spectrometer (Thermo Fisher Scientific). The quality of total RNAs was checked by an Agilent 2100 BioAnalyzer (Agilent). Each cDNA with T7 promoter-tag was synthesized from 500 ng of total RNA according to the Agilent's protocol. cRNA labelled with a fluorescent dye (Cy3-CTP) was produced according to the Agilent's protocol and then purified with an RNeasy column (QIAGEN). Before loading onto a microarray, each labelled cRNA was fragmented in hybridization buffer at 60°C within 30 min. The array was hybridized with labelled cRNA at 65°C for 17 h and washed by Agilent Gene Expression Wash Buffer 1 and 2, respectively. All microarrays were scanned using an Agilent Microarray Scanner (Agilent). The signal intensity of each spot was measured using Agilent Feature Extraction version 9.5 (Agilent). These data were converted Excel (Microsoft) file, and then analyzed.

## Supporting Information

Figure S1 **Cellular response to 20E of two** ***Bombyx*** **cultured cells and the 20E-dependent expression of three nuclear receptor genes.** (A) A *Bombyx* cell line aff3 showed an aggregated cellular response (white arrows) to 2.0×10^−2^ and 2.0 µg/mL of 20E, whereas BmN did not show a remarkable cellular response to 20E. (B) Time course of 20E-dependent expression patterns of *BmEcR-B1*, *BmEcR-A*, *BmE75-A* and *BHR3-B* in aff3 cells analyzed by Northern hybridization. Total RNA was extracted from the cells incubated with 2.0 µg/mL of 20E in the medium at 3, 6, 12 and 24 h. rRNA stained by ethidium bromide is shown as equal loading. (C) Dose dependency of *BmEcR-B1*, *BmE75-A*, and *BHR3-B* expressions in the response to 20E were analyzed in aff3 cells. The conditions for Northern hybridization were same as (B). The probes for *BmEcR-B1* and *BmE75-A* were hybridized to the same membrane, transferred the total RNAs extracting from aff3 cells incubated 3 hrs with each diluted 20E. BHR3-B was detected from another membrane, incubating 12 h with 20Es.
(TIF)

Figure S2 **Identification of the ecdysone responsive region in** ***BmEcR-B1*** **promoter.** (A–C) Each promoter activity of 5′-deletion series (A) and excision series (B, C) of constructs for the *BmEcR-B1* promoter region is shown. Each plasmid construct was transfected into aff3 cells and incubated 48 h with 2.0 µg/mL (4.2 µM) of 20E and measured the luciferase activity. The ratio of relative luciferase activities with and without 20E (fold induction by 20E) is shown on the right, as referred 1.0 at 48 h without 20E. Error bar represents SE (N = 4). “null” indicates the pGL4.10 vector. (B, C) Excisions are shown by the white squares. The sizes of the excisions are 40 bp (B) and 10 bp (C), respectively.
(TIF)

Figure S3 **Identification of EcREs for** ***BmE75-A*** **.** (A, B) Each promoter activity of 5′-deletion series of constructs for *BmE75-A* promoter region is shown. The fold induction by 20E of each construct is shown in right. Error bars (N = 4). “Null”; the pGL4.10 vector.
(TIF)

Figure S4 **Identification of EcREs for** ***BHR3-B*** **.** Each promoter activity of 5′-deletion series of constructs for *BHR3-B* promoter region is shown. The fold induction by 20E of each construct is shown in right. Error bars (N = 4). “Null”; the pGL4.10 vector.
(TIF)

Figure S5 **Effects of swapping EcRE between** ***BmE75-A*** **and** ***BHR3-B*** **promoter regions on their responses to 20E.** (A) Schematic structures of each reporter construct for EcRE swapping between *BmE75-A* and *BHR3-B* promoter regions. EcREs of *BmE75-A* and *BHR3-B* full-length reporter plasmids were replaced with each other. BHR3B-EcRE, TCGGGTCAACGAACCGGTGT; BmE75A-EcRE, TCGGGTCTTCGAACTCTCGG (B) Dose response to 20E of each construct. Each reporter plasmid was transfected into the aff3 cell and incubated with various concentrations of 20E for 2 days. The reporter activities were measured by a dual-luciferase assay. Bars represent SE (N = 6).
(TIF)

Figure S6 **Electrophoretic mobility shift analysis for BmE75A-EcRE and BHR3B-EcRE.** (A, B) Competition assay with cold probe for BmE75A-EcRE (A) and for BHR3B-EcRE (B). Mutation sites in E1 and E2 probe sequence “Normal” are shown in the gray region of “Mutant”. Two hundred femtomoles of ^32^P-probe were incubated with 5 µg of cell extracts and loaded onto the gel. 20E (6 h) represents extracts from cells cultured under 20E during 6 h. ×1, ×10, ×50 and ×100 represent the ratio of the cold probe amount to the ^32^P-probe amount. Filled arrows show the shifted bands and blank arrows show the free probes. (C, D) Super shift assay with anti-V5 or/and anti-USP antibodies for BmE75A-EcRE (C) and for BHR3B-EcRE (D). Intact: intact cell extracts. A^T^, B1^T^, and USP^T^ represent extracts from cells that overexpressed EcRA, EcRB1, and USP, respectively. (E) Western blot analysis of the overexpressed nuclear receptors. Each protein with a V5-tag was detected by the anti-V5 antibody.
(TIF)

Figure S7 Conservation of EcREs for *HR3-B* (A) and *E75-A* (B) in Lepidoptera. (A) Highly homologous sequences, BHR3B-EcRE and EcRE1 of *Manduca HR3* (MHR3-EcRE1) and its similar sequences, are shown by red and yellow boxes in the upper section, respectively. Three highly conserved regions, A, B, and C between *BHR3* and *MHR3* promoter regions shown by blue thick lines. EcRE2, 3, and 4, which were identified in *Manduca MHR3*, are shown by open box. The homologous sequences to *Manduca* EcRE2, 3, and 4 are not found in the promoter region of *BHR3*. +1, the transcriptional start site. Shaded box, the *HR3* gene. Sequence comparison of each element is shown in the lower section. *B. m, Bombyx mori; M. s, Manduca sexta; D. p, Danaus plexippus; P. x, Papilio xuthus*. Nucleotide sequences in the region A (yellow, the same sequence to *B mori* EcRE-like element) and the region B (red, the same sequence to BHR3-B EcRE) are aligned. *, a consensus nucleotide among the sequences. (B) Schematic localization of *E75-A* of four lepidopteran insects (upper section) and sequence comparison of each element (lower section). Red, the same sequence to BmE75-A EcRE. *, a consensus nucleotide among the sequences. ATG: the translational start site.
(TIF)

Table S1 List of Accession Number.
(PPT)

Table S2 List of Primer.
(PPT)

Table S3 List of Primer.
(PPT)

Table S4 List of Primer.
(PPT)

Table S5 List of Primer.
(PPT)

Table S6 List of 20E-inducible genes identified by microarray analysis and the 14 bp consensus motifs.
(PPT)
