# Supplementary figures and images for: Two Adjacent cis-Regulatory Elements Are Required for Ecdysone Response of Ecdysone Receptor (EcR) B1 Transcription
Source: PLoS One. 2012 Nov 14;7(11):e49348. doi: 10.1371/journal.pone.0049348 (PMC3498158; doi:10.1371/journal.pone.0049348)

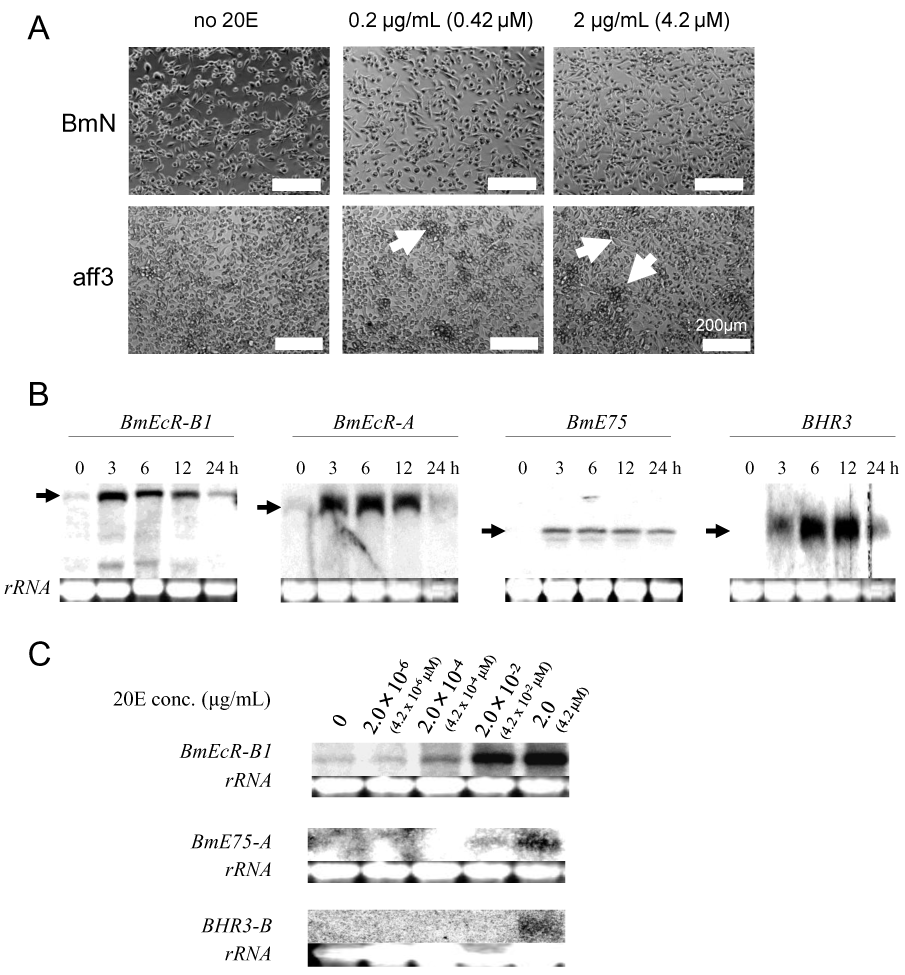

Supplement: Figure S1 — Cellular response to 20E of two Bombyx cultured cells and the 20E-dependent expression of three nuclear receptor genes. (A) A Bombyx cell line aff3 showed an aggregated cellular response (white arrows) to 2.0×10^−2^ and 2.0 µg/mL of 20E, whereas BmN did not show a remarkable cellular response to 20E. (B) Time course of 20E-dependent expression patterns of BmEcR-B1, BmEcR-A, BmE75-A and BHR3-B in aff3 cells analyzed by Northern hybridization. Total RNA was extracted from the cells incubated with 2.0 µg/mL of 20E in the medium at 3, 6, 12 and 24 h. rRNA stained by ethidium bromide is shown as equal loading. (C) Dose dependency of BmEcR-B1, BmE75-A, and BHR3-B expressions in the response to 20E were analyzed in aff3 cells. The conditions for Northern hybridization were same as (B). The probes for BmEcR-B1 and BmE75-A were hybridized to the same membrane, transferred the total RNAs extracting from aff3 cells incubated 3 hrs with each diluted 20E. BHR3-B was detected from another membrane, incubating 12 h with 20Es. (TIF) [file pone.0049348.s001.tif]

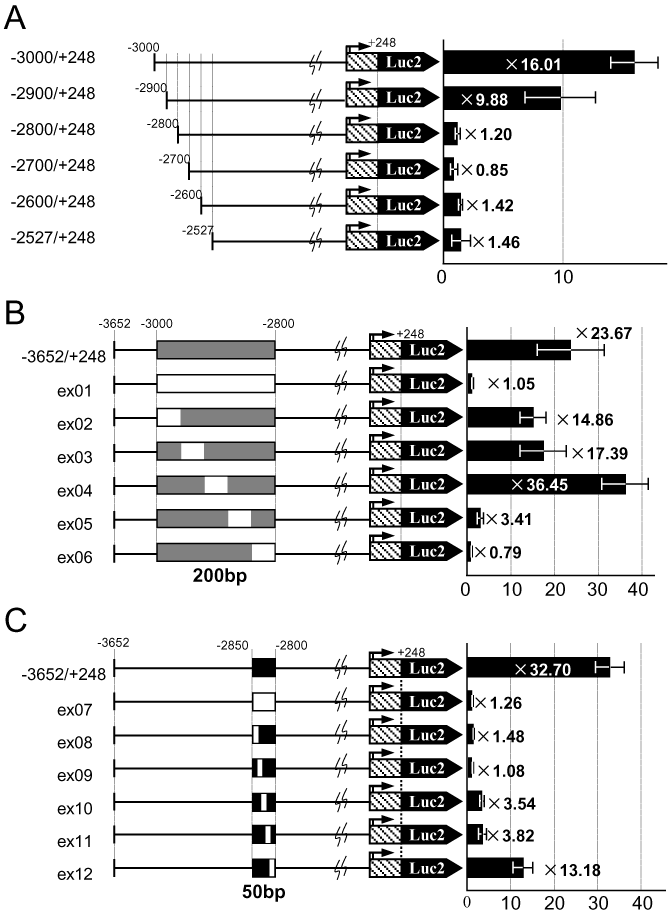

Supplement: Figure S2 — Identification of the ecdysone responsive region in BmEcR-B1 promoter. (A–C) Each promoter activity of 5′-deletion series (A) and excision series (B, C) of constructs for the BmEcR-B1 promoter region is shown. Each plasmid construct was transfected into aff3 cells and incubated 48 h with 2.0 µg/mL (4.2 µM) of 20E and measured the luciferase activity. The ratio of relative luciferase activities with and without 20E (fold induction by 20E) is shown on the right, as referred 1.0 at 48 h without 20E. Error bar represents SE (N = 4). “null” indicates the pGL4.10 vector. (B, C) Excisions are shown by the white squares. The sizes of the excisions are 40 bp (B) and 10 bp (C), respectively. (TIF) [file pone.0049348.s002.tif]

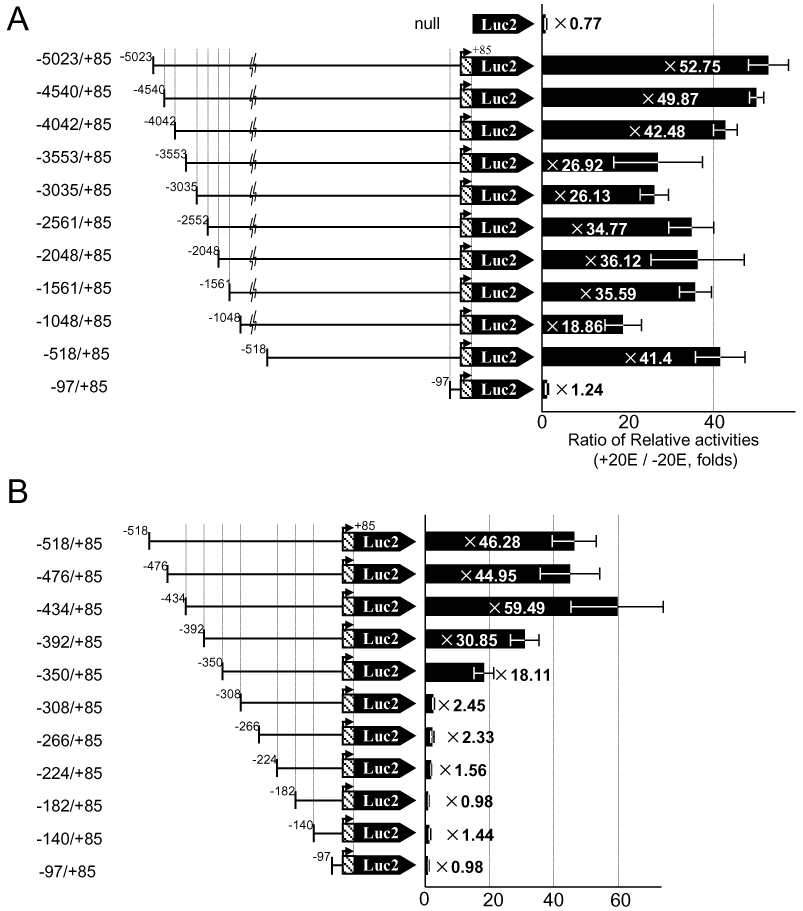

Supplement: Figure S3 — Identification of EcREs for BmE75-A . (A, B) Each promoter activity of 5′-deletion series of constructs for BmE75-A promoter region is shown. The fold induction by 20E of each construct is shown in right. Error bars (N = 4). “Null”; the pGL4.10 vector. (TIF) [file pone.0049348.s003.tif]

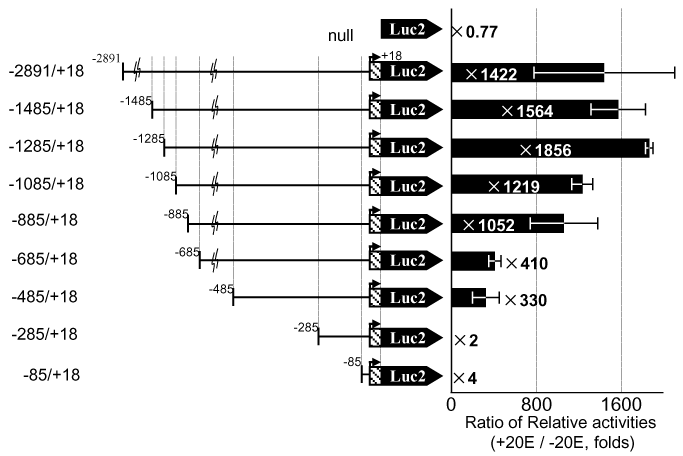

Supplement: Figure S4 — Identification of EcREs for BHR3-B . Each promoter activity of 5′-deletion series of constructs for BHR3-B promoter region is shown. The fold induction by 20E of each construct is shown in right. Error bars (N = 4). “Null”; the pGL4.10 vector. (TIF) [file pone.0049348.s004.tif]

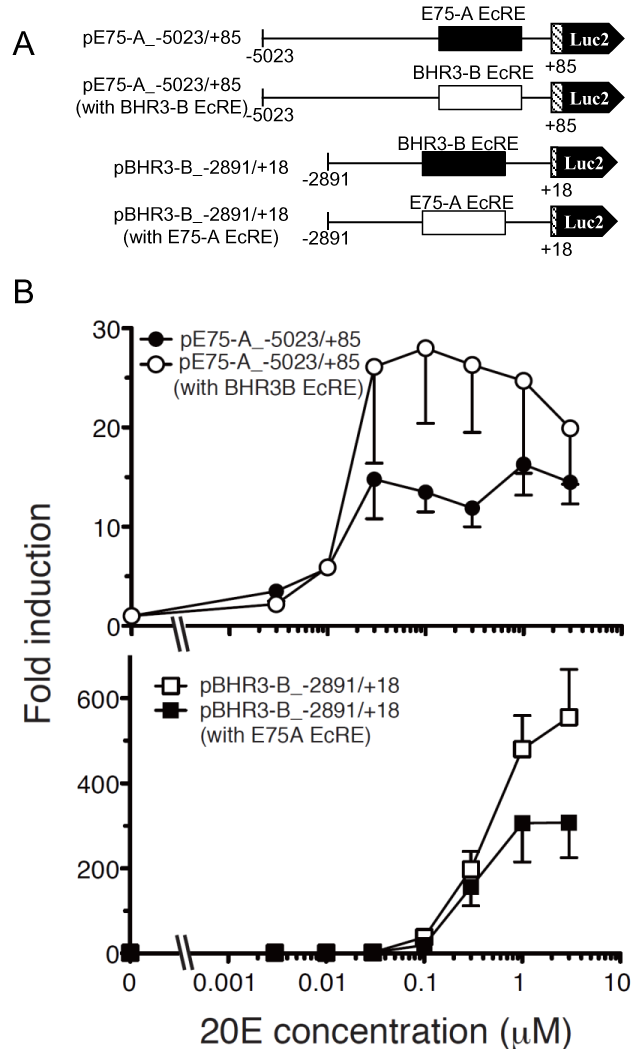

Supplement: Figure S5 — Effects of swapping EcRE between BmE75-A and BHR3-B promoter regions on their responses to 20E. (A) Schematic structures of each reporter construct for EcRE swapping between BmE75-A and BHR3-B promoter regions. EcREs of BmE75-A and BHR3-B full-length reporter plasmids were replaced with each other. BHR3B-EcRE, TCGGGTCAACGAACCGGTGT; BmE75A-EcRE, TCGGGTCTTCGAACTCTCGG (B) Dose response to 20E of each construct. Each reporter plasmid was transfected into the aff3 cell and incubated with various concentrations of 20E for 2 days. The reporter activities were measured by a dual-luciferase assay. Bars represent SE (N = 6). (TIF) [file pone.0049348.s005.tif]

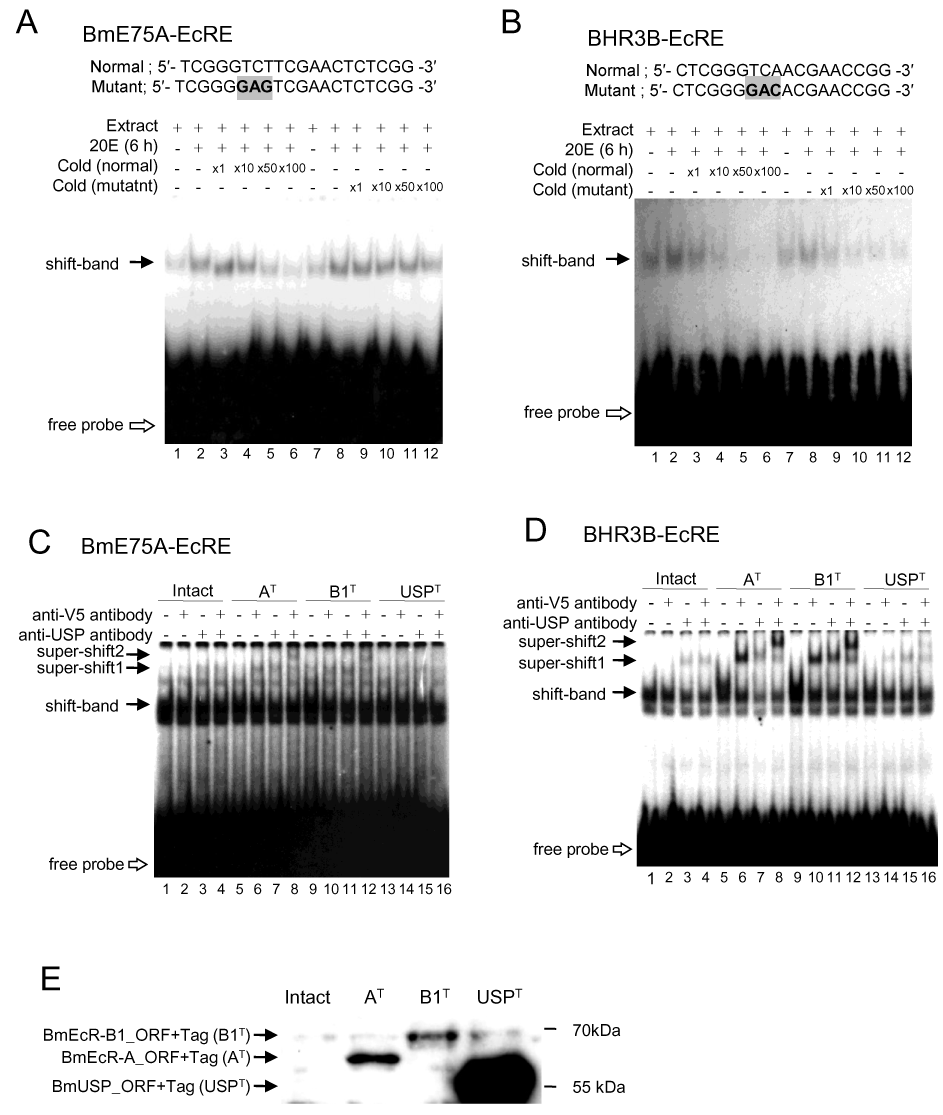

Supplement: Figure S6 — Electrophoretic mobility shift analysis for BmE75A-EcRE and BHR3B-EcRE. (A, B) Competition assay with cold probe for BmE75A-EcRE (A) and for BHR3B-EcRE (B). Mutation sites in E1 and E2 probe sequence “Normal” are shown in the gray region of “Mutant”. Two hundred femtomoles of 32P-probe were incubated with 5 µg of cell extracts and loaded onto the gel. 20E (6 h) represents extracts from cells cultured under 20E during 6 h. ×1, ×10, ×50 and ×100 represent the ratio of the cold probe amount to the 32P-probe amount. Filled arrows show the shifted bands and blank arrows show the free probes. (C, D) Super shift assay with anti-V5 or/and anti-USP antibodies for BmE75A-EcRE (C) and for BHR3B-EcRE (D). Intact: intact cell extracts. AT, B1T, and USPT represent extracts from cells that overexpressed EcRA, EcRB1, and USP, respectively. (E) Western blot analysis of the overexpressed nuclear receptors. Each protein with a V5-tag was detected by the anti-V5 antibody. (TIF) [file pone.0049348.s006.tif]

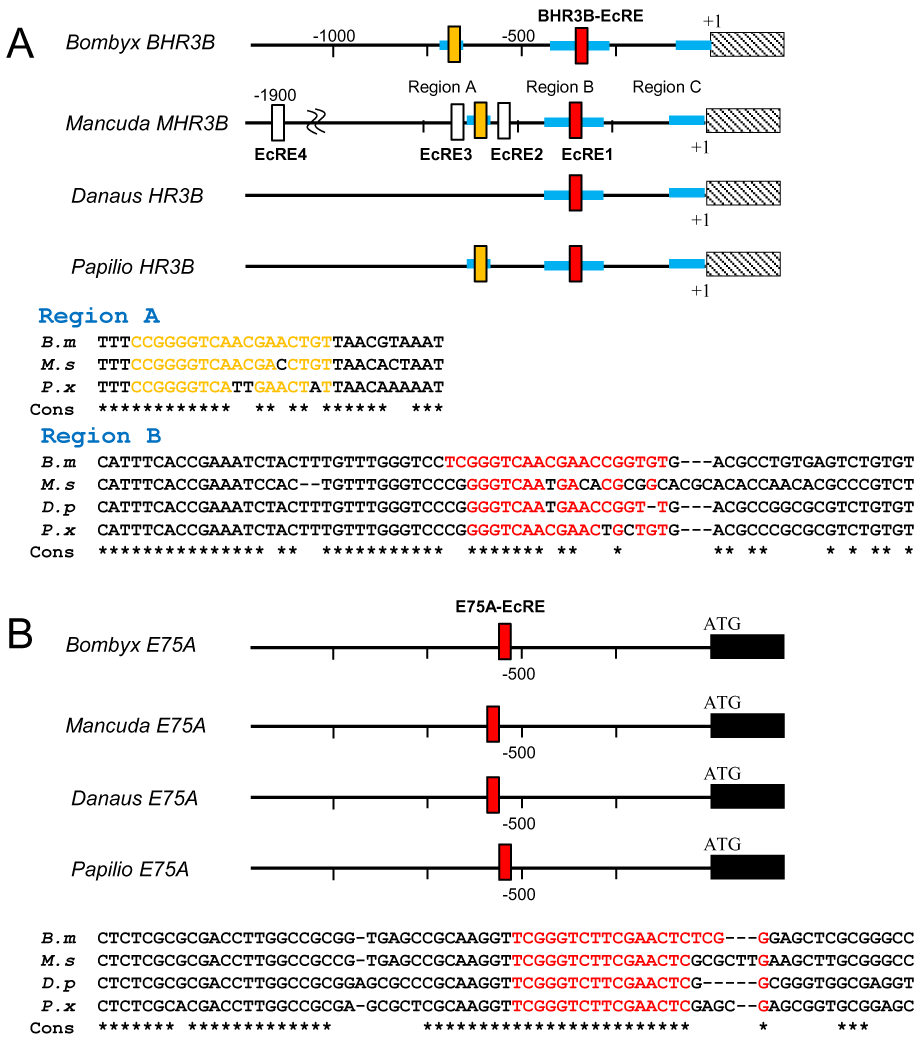

Supplement: Figure S7 — Conservation of EcREs for HR3-B (A) and E75-A (B) in Lepidoptera. (A) Highly homologous sequences, BHR3B-EcRE and EcRE1 of Manduca HR3 (MHR3-EcRE1) and its similar sequences, are shown by red and yellow boxes in the upper section, respectively. Three highly conserved regions, A, B, and C between BHR3 and MHR3 promoter regions shown by blue thick lines. EcRE2, 3, and 4, which were identified in Manduca MHR3, are shown by open box. The homologous sequences to Manduca EcRE2, 3, and 4 are not found in the promoter region of BHR3. +1, the transcriptional start site. Shaded box, the HR3 gene. Sequence comparison of each element is shown in the lower section. B. m, Bombyx mori; M. s, Manduca sexta; D. p, Danaus plexippus; P. x, Papilio xuthus. Nucleotide sequences in the region A (yellow, the same sequence to B mori EcRE-like element) and the region B (red, the same sequence to BHR3-B EcRE) are aligned. *, a consensus nucleotide among the sequences. (B) Schematic localization of E75-A of four lepidopteran insects (upper section) and sequence comparison of each element (lower section). Red, the same sequence to BmE75-A EcRE. *, a consensus nucleotide among the sequences. ATG: the translational start site. (TIF) [file pone.0049348.s007.tif]
