# Supplementary material for: Two Adjacent cis-Regulatory Elements Are Required for Ecdysone Response of Ecdysone Receptor (EcR) B1 Transcription
Source: PLoS One. 2012 Nov 14;7(11):e49348. doi: 10.1371/journal.pone.0049348 (PMC3498158; doi:10.1371/journal.pone.0049348)
Supplement: Table S1 — List of Accession Number. (PPT) [file pone.0049348.s008.ppt]

## Slide 1
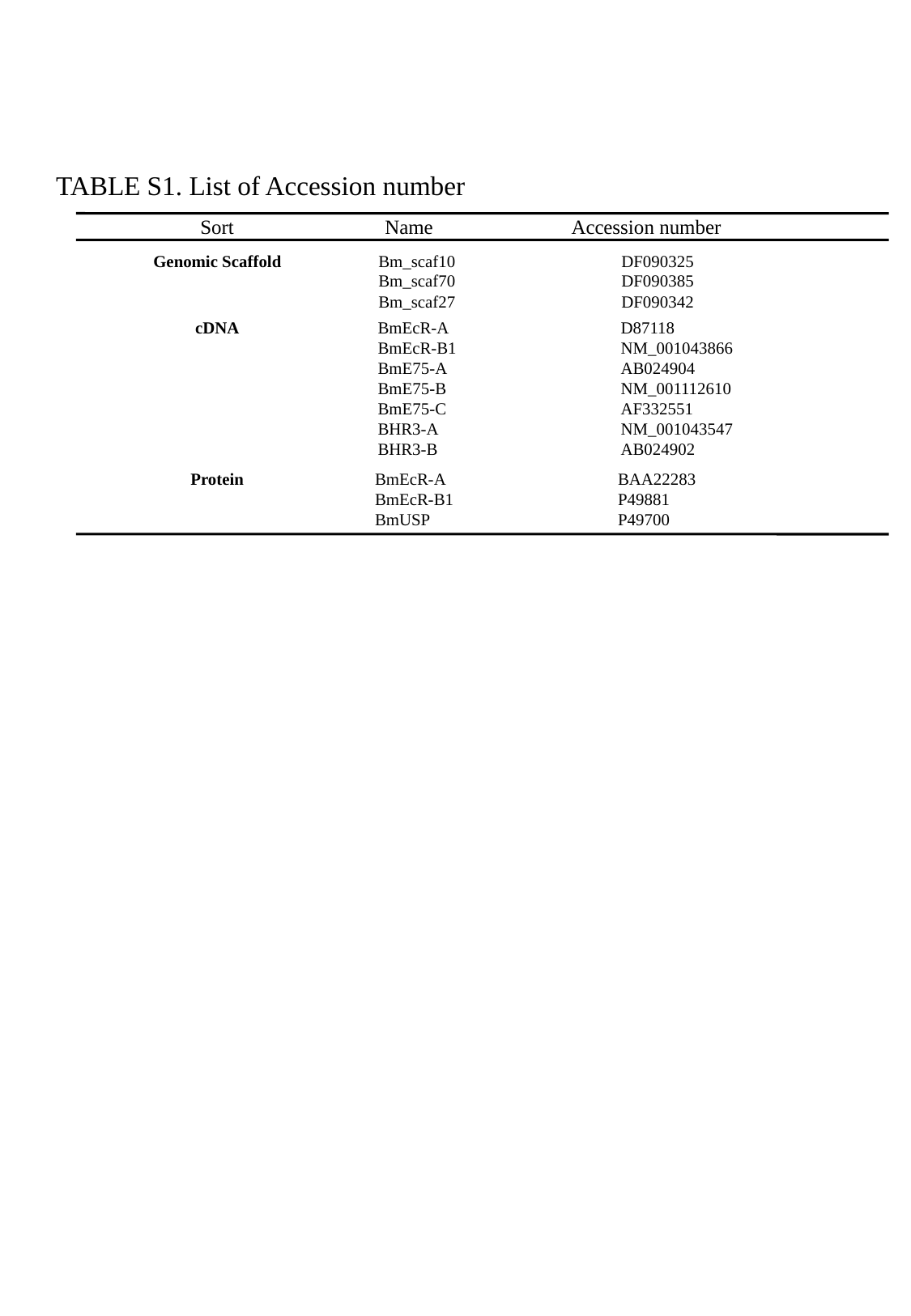

TABLE S1. List of Accession number
Accession number
Sort
Name
Genomic Scaffold
Bm_scaf10		DF090325
Bm_scaf70		DF090385
Bm_scaf27		DF090342
cDNA
BmEcR-A		D87118
BmEcR-B1		NM_001043866
BmE75-A		AB024904
BmE75-B		NM_001112610
BmE75-C		AF332551
BHR3-A		NM_001043547
BHR3-B		AB024902
Protein
BmEcR-A		BAA22283
BmEcR-B1		P49881
BmUSP		P49700
