# Supplementary material for: Two Adjacent cis-Regulatory Elements Are Required for Ecdysone Response of Ecdysone Receptor (EcR) B1 Transcription
Source: PLoS One. 2012 Nov 14;7(11):e49348. doi: 10.1371/journal.pone.0049348 (PMC3498158; doi:10.1371/journal.pone.0049348)
Supplement: Table S2 — List of Primer. (PPT) [file pone.0049348.s009.ppt]

## Slide 1
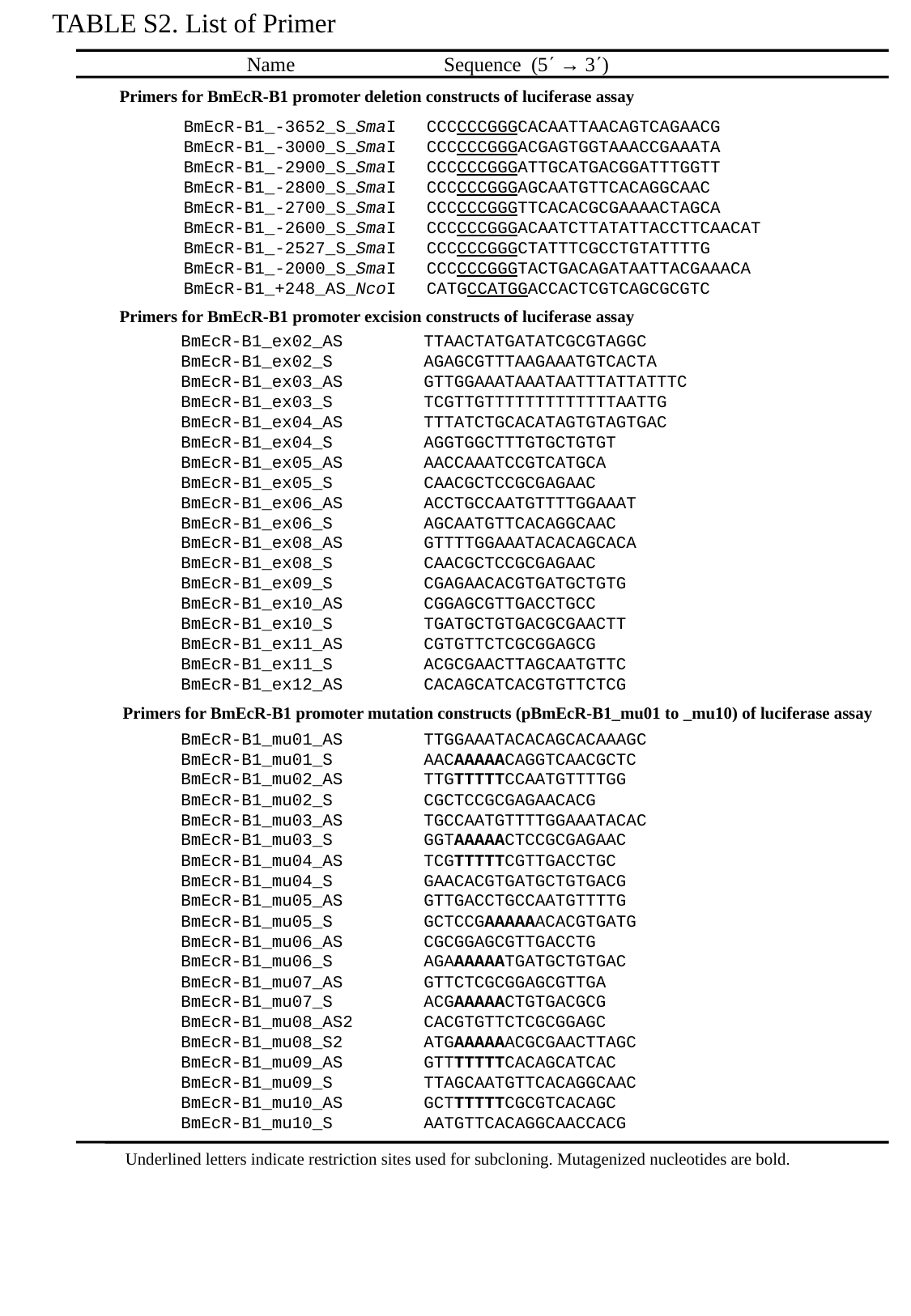

TABLE S2. List of Primer
Name
Sequence (5′ → 3′)
Primers for BmEcR-B1 promoter deletion constructs of luciferase assay
BmEcR-B1_-3652_S_SmaI	CCCCCCGGGCACAATTAACAGTCAGAACG
BmEcR-B1_-3000_S_SmaI	CCCCCCGGGACGAGTGGTAAACCGAAATA
BmEcR-B1_-2900_S_SmaI	CCCCCCGGGATTGCATGACGGATTTGGTT
BmEcR-B1_-2800_S_SmaI	CCCCCCGGGAGCAATGTTCACAGGCAAC
BmEcR-B1_-2700_S_SmaI	CCCCCCGGGTTCACACGCGAAAACTAGCA
BmEcR-B1_-2600_S_SmaI	CCCCCCGGGACAATCTTATATTACCTTCAACAT
BmEcR-B1_-2527_S_SmaI	CCCCCCGGGCTATTTCGCCTGTATTTTG
BmEcR-B1_-2000_S_SmaI	CCCCCCGGGTACTGACAGATAATTACGAAACA
BmEcR-B1_+248_AS_NcoI	CATGCCATGGACCACTCGTCAGCGCGTC
Primers for BmEcR-B1 promoter excision constructs of luciferase assay
BmEcR-B1_ex02_AS	TTAACTATGATATCGCGTAGGC
BmEcR-B1_ex02_S	AGAGCGTTTAAGAAATGTCACTA
BmEcR-B1_ex03_AS	GTTGGAAATAAATAATTTATTATTTC
BmEcR-B1_ex03_S	TCGTTGTTTTTTTTTTTTTAATTG
BmEcR-B1_ex04_AS	TTTATCTGCACATAGTGTAGTGAC
BmEcR-B1_ex04_S	AGGTGGCTTTGTGCTGTGT
BmEcR-B1_ex05_AS	AACCAAATCCGTCATGCA
BmEcR-B1_ex05_S	CAACGCTCCGCGAGAAC
BmEcR-B1_ex06_AS	ACCTGCCAATGTTTTGGAAAT
BmEcR-B1_ex06_S	AGCAATGTTCACAGGCAAC
BmEcR-B1_ex08_AS	GTTTTGGAAATACACAGCACA
BmEcR-B1_ex08_S	CAACGCTCCGCGAGAAC
BmEcR-B1_ex09_S	CGAGAACACGTGATGCTGTG
BmEcR-B1_ex10_AS	CGGAGCGTTGACCTGCC
BmEcR-B1_ex10_S	TGATGCTGTGACGCGAACTT
BmEcR-B1_ex11_AS	CGTGTTCTCGCGGAGCG
BmEcR-B1_ex11_S	ACGCGAACTTAGCAATGTTC
BmEcR-B1_ex12_AS	CACAGCATCACGTGTTCTCG
Primers for BmEcR-B1 promoter mutation constructs (pBmEcR-B1_mu01 to _mu10) of luciferase assay
BmEcR-B1_mu01_AS	TTGGAAATACACAGCACAAAGC
BmEcR-B1_mu01_S	AACAAAAACAGGTCAACGCTC
BmEcR-B1_mu02_AS	TTGTTTTTCCAATGTTTTGG
BmEcR-B1_mu02_S	CGCTCCGCGAGAACACG
BmEcR-B1_mu03_AS	TGCCAATGTTTTGGAAATACAC
BmEcR-B1_mu03_S	GGTAAAAACTCCGCGAGAAC
BmEcR-B1_mu04_AS	TCGTTTTTCGTTGACCTGC
BmEcR-B1_mu04_S	GAACACGTGATGCTGTGACG
BmEcR-B1_mu05_AS	GTTGACCTGCCAATGTTTTG
BmEcR-B1_mu05_S	GCTCCGAAAAAACACGTGATG
BmEcR-B1_mu06_AS	CGCGGAGCGTTGACCTG
BmEcR-B1_mu06_S	AGAAAAAATGATGCTGTGAC
BmEcR-B1_mu07_AS	GTTCTCGCGGAGCGTTGA
BmEcR-B1_mu07_S	ACGAAAAACTGTGACGCG
BmEcR-B1_mu08_AS2	CACGTGTTCTCGCGGAGC
BmEcR-B1_mu08_S2	ATGAAAAAACGCGAACTTAGC
BmEcR-B1_mu09_AS	GTTTTTTTCACAGCATCAC
BmEcR-B1_mu09_S	TTAGCAATGTTCACAGGCAAC
BmEcR-B1_mu10_AS	GCTTTTTTCGCGTCACAGC
BmEcR-B1_mu10_S	AATGTTCACAGGCAACCACG
Underlined letters indicate restriction sites used for subcloning. Mutagenized nucleotides are bold.
