# Supplementary material for: Two Adjacent cis-Regulatory Elements Are Required for Ecdysone Response of Ecdysone Receptor (EcR) B1 Transcription
Source: PLoS One. 2012 Nov 14;7(11):e49348. doi: 10.1371/journal.pone.0049348 (PMC3498158; doi:10.1371/journal.pone.0049348)
Supplement: Table S3 — List of Primer. (PPT) [file pone.0049348.s010.ppt]

## Slide 1
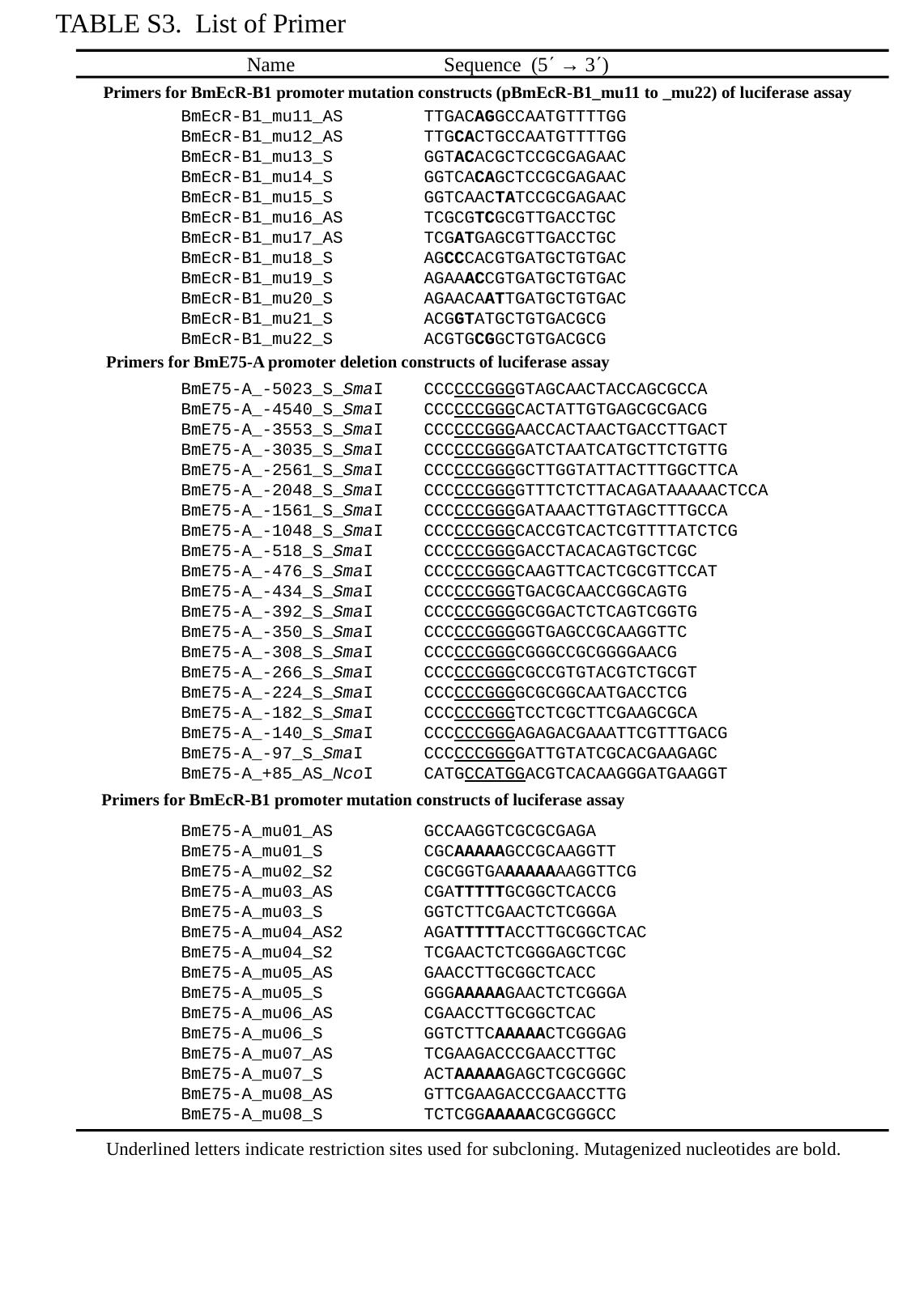

TABLE S3. List of Primer
Name
Sequence (5′ → 3′)
Primers for BmEcR-B1 promoter mutation constructs (pBmEcR-B1_mu11 to _mu22) of luciferase assay
BmEcR-B1_mu11_AS	TTGACAGGCCAATGTTTTGG
BmEcR-B1_mu12_AS	TTGCACTGCCAATGTTTTGG
BmEcR-B1_mu13_S	GGTACACGCTCCGCGAGAAC
BmEcR-B1_mu14_S	GGTCACAGCTCCGCGAGAAC
BmEcR-B1_mu15_S	GGTCAACTATCCGCGAGAAC
BmEcR-B1_mu16_AS	TCGCGTCGCGTTGACCTGC
BmEcR-B1_mu17_AS	TCGATGAGCGTTGACCTGC
BmEcR-B1_mu18_S	AGCCCACGTGATGCTGTGAC
BmEcR-B1_mu19_S	AGAAACCGTGATGCTGTGAC
BmEcR-B1_mu20_S	AGAACAATTGATGCTGTGAC
BmEcR-B1_mu21_S	ACGGTATGCTGTGACGCG
BmEcR-B1_mu22_S	ACGTGCGGCTGTGACGCG
Primers for BmE75-A promoter deletion constructs of luciferase assay
BmE75-A_-5023_S_SmaI	CCCCCCGGGGTAGCAACTACCAGCGCCA
BmE75-A_-4540_S_SmaI	CCCCCCGGGCACTATTGTGAGCGCGACG
BmE75-A_-3553_S_SmaI	CCCCCCGGGAACCACTAACTGACCTTGACT
BmE75-A_-3035_S_SmaI	CCCCCCGGGGATCTAATCATGCTTCTGTTG
BmE75-A_-2561_S_SmaI	CCCCCCGGGGCTTGGTATTACTTTGGCTTCA
BmE75-A_-2048_S_SmaI	CCCCCCGGGGTTTCTCTTACAGATAAAAACTCCA
BmE75-A_-1561_S_SmaI	CCCCCCGGGGATAAACTTGTAGCTTTGCCA
BmE75-A_-1048_S_SmaI	CCCCCCGGGCACCGTCACTCGTTTTATCTCG
BmE75-A_-518_S_SmaI	CCCCCCGGGGACCTACACAGTGCTCGC
BmE75-A_-476_S_SmaI	CCCCCCGGGCAAGTTCACTCGCGTTCCAT
BmE75-A_-434_S_SmaI	CCCCCCGGGTGACGCAACCGGCAGTG
BmE75-A_-392_S_SmaI	CCCCCCGGGGCGGACTCTCAGTCGGTG
BmE75-A_-350_S_SmaI	CCCCCCGGGGGTGAGCCGCAAGGTTC
BmE75-A_-308_S_SmaI	CCCCCCGGGCGGGCCGCGGGGAACG
BmE75-A_-266_S_SmaI	CCCCCCGGGCGCCGTGTACGTCTGCGT
BmE75-A_-224_S_SmaI	CCCCCCGGGGCGCGGCAATGACCTCG
BmE75-A_-182_S_SmaI	CCCCCCGGGTCCTCGCTTCGAAGCGCA
BmE75-A_-140_S_SmaI	CCCCCCGGGAGAGACGAAATTCGTTTGACG
BmE75-A_-97_S_SmaI	CCCCCCGGGGATTGTATCGCACGAAGAGC
BmE75-A_+85_AS_NcoI	CATGCCATGGACGTCACAAGGGATGAAGGT
Primers for BmEcR-B1 promoter mutation constructs of luciferase assay
BmE75-A_mu01_AS	GCCAAGGTCGCGCGAGA
BmE75-A_mu01_S	CGCAAAAAGCCGCAAGGTT
BmE75-A_mu02_S2	CGCGGTGAAAAAAAAGGTTCG
BmE75-A_mu03_AS	CGATTTTTGCGGCTCACCG
BmE75-A_mu03_S	GGTCTTCGAACTCTCGGGA
BmE75-A_mu04_AS2	AGATTTTTACCTTGCGGCTCAC
BmE75-A_mu04_S2	TCGAACTCTCGGGAGCTCGC
BmE75-A_mu05_AS	GAACCTTGCGGCTCACC
BmE75-A_mu05_S	GGGAAAAAGAACTCTCGGGA
BmE75-A_mu06_AS	CGAACCTTGCGGCTCAC
BmE75-A_mu06_S	GGTCTTCAAAAACTCGGGAG
BmE75-A_mu07_AS	TCGAAGACCCGAACCTTGC
BmE75-A_mu07_S	ACTAAAAAGAGCTCGCGGGC
BmE75-A_mu08_AS	GTTCGAAGACCCGAACCTTG
BmE75-A_mu08_S	TCTCGGAAAAACGCGGGCC
Underlined letters indicate restriction sites used for subcloning. Mutagenized nucleotides are bold.
