# Supplementary material for: Two Adjacent cis-Regulatory Elements Are Required for Ecdysone Response of Ecdysone Receptor (EcR) B1 Transcription
Source: PLoS One. 2012 Nov 14;7(11):e49348. doi: 10.1371/journal.pone.0049348 (PMC3498158; doi:10.1371/journal.pone.0049348)
Supplement: Table S4 — List of Primer. (PPT) [file pone.0049348.s011.ppt]

## Slide 1
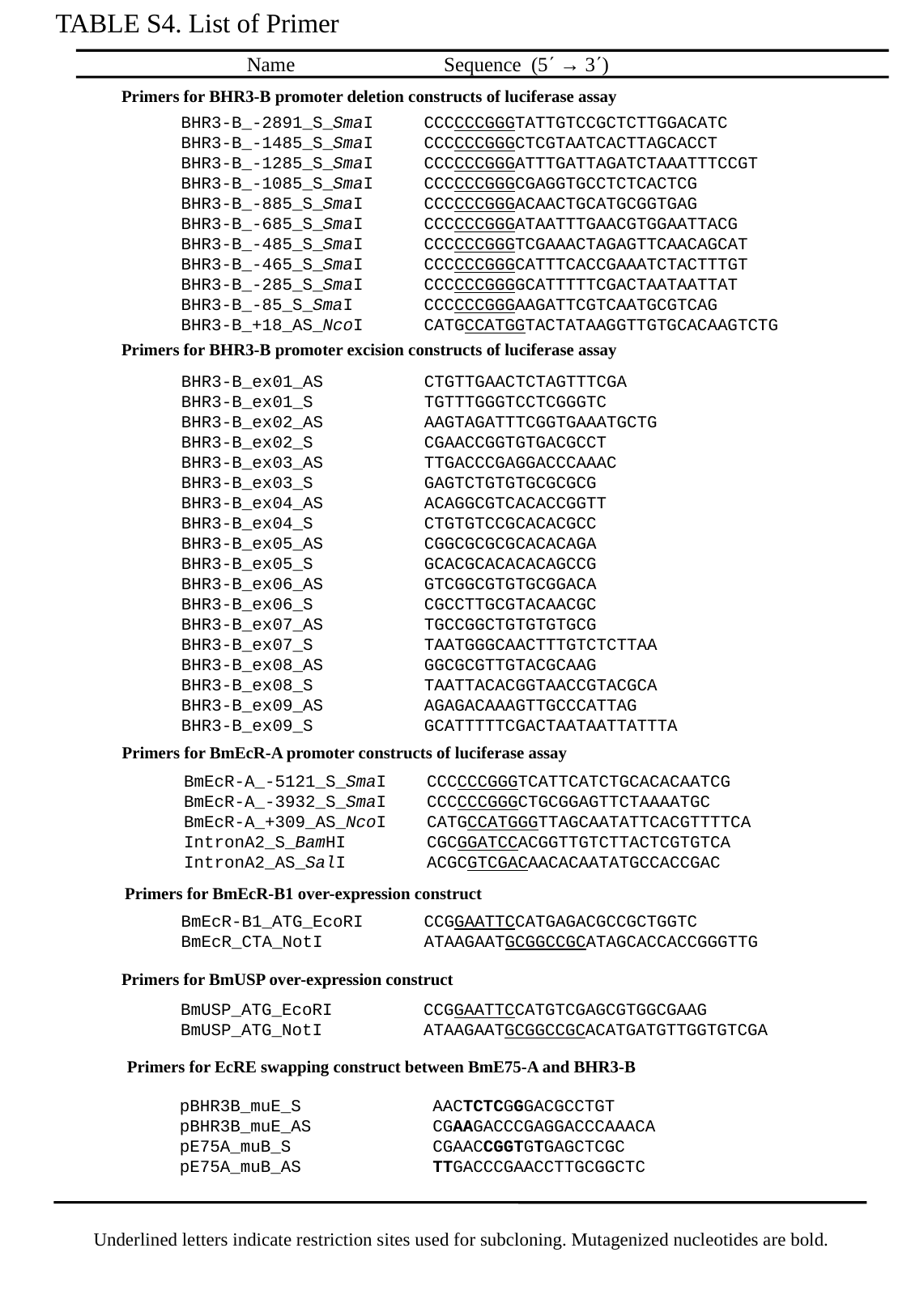

TABLE S4. List of Primer
Name
Sequence (5′ → 3′)
Primers for BHR3-B promoter deletion constructs of luciferase assay
BHR3-B_-2891_S_SmaI	CCCCCCGGGTATTGTCCGCTCTTGGACATC
BHR3-B_-1485_S_SmaI	CCCCCCGGGCTCGTAATCACTTAGCACCT
BHR3-B_-1285_S_SmaI	CCCCCCGGGATTTGATTAGATCTAAATTTCCGT
BHR3-B_-1085_S_SmaI	CCCCCCGGGCGAGGTGCCTCTCACTCG
BHR3-B_-885_S_SmaI	CCCCCCGGGACAACTGCATGCGGTGAG
BHR3-B_-685_S_SmaI	CCCCCCGGGATAATTTGAACGTGGAATTACG
BHR3-B_-485_S_SmaI	CCCCCCGGGTCGAAACTAGAGTTCAACAGCAT
BHR3-B_-465_S_SmaI	CCCCCCGGGCATTTCACCGAAATCTACTTTGT
BHR3-B_-285_S_SmaI	CCCCCCGGGGCATTTTTCGACTAATAATTAT
BHR3-B_-85_S_SmaI	CCCCCCGGGAAGATTCGTCAATGCGTCAG
BHR3-B_+18_AS_NcoI	CATGCCATGGTACTATAAGGTTGTGCACAAGTCTG
Primers for BHR3-B promoter excision constructs of luciferase assay
BHR3-B_ex01_AS	CTGTTGAACTCTAGTTTCGA
BHR3-B_ex01_S	TGTTTGGGTCCTCGGGTC
BHR3-B_ex02_AS	AAGTAGATTTCGGTGAAATGCTG
BHR3-B_ex02_S	CGAACCGGTGTGACGCCT
BHR3-B_ex03_AS	TTGACCCGAGGACCCAAAC
BHR3-B_ex03_S	GAGTCTGTGTGCGCGCG
BHR3-B_ex04_AS	ACAGGCGTCACACCGGTT
BHR3-B_ex04_S	CTGTGTCCGCACACGCC
BHR3-B_ex05_AS	CGGCGCGCGCACACAGA
BHR3-B_ex05_S	GCACGCACACACAGCCG
BHR3-B_ex06_AS	GTCGGCGTGTGCGGACA
BHR3-B_ex06_S	CGCCTTGCGTACAACGC
BHR3-B_ex07_AS	TGCCGGCTGTGTGTGCG
BHR3-B_ex07_S	TAATGGGCAACTTTGTCTCTTAA
BHR3-B_ex08_AS	GGCGCGTTGTACGCAAG
BHR3-B_ex08_S	TAATTACACGGTAACCGTACGCA
BHR3-B_ex09_AS	AGAGACAAAGTTGCCCATTAG
BHR3-B_ex09_S	GCATTTTTCGACTAATAATTATTTA
Primers for BmEcR-A promoter constructs of luciferase assay
BmEcR-A_-5121_S_SmaI	CCCCCCGGGTCATTCATCTGCACACAATCG
BmEcR-A_-3932_S_SmaI CCCCCCGGGCTGCGGAGTTCTAAAATGC
BmEcR-A_+309_AS_NcoI CATGCCATGGGTTAGCAATATTCACGTTTTCA
IntronA2_S_BamHI	CGCGGATCCACGGTTGTCTTACTCGTGTCA
IntronA2_AS_SalI	ACGCGTCGACAACACAATATGCCACCGAC
Primers for BmEcR-B1 over-expression construct
BmEcR-B1_ATG_EcoRI	CCGGAATTCCATGAGACGCCGCTGGTC
BmEcR_CTA_NotI	ATAAGAATGCGGCCGCATAGCACCACCGGGTTG
Primers for BmUSP over-expression construct
BmUSP_ATG_EcoRI	CCGGAATTCCATGTCGAGCGTGGCGAAG
BmUSP_ATG_NotI	ATAAGAATGCGGCCGCACATGATGTTGGTGTCGA
Primers for EcRE swapping construct between BmE75-A and BHR3-B
pBHR3B_muE_S	 AACTCTCGGGACGCCTGT
pBHR3B_muE_AS	 CGAAGACCCGAGGACCCAAACA
pE75A_muB_S 	 CGAACCGGTGTGAGCTCGC
pE75A_muB_AS	 TTGACCCGAACCTTGCGGCTC
Underlined letters indicate restriction sites used for subcloning. Mutagenized nucleotides are bold.
