# Supplementary material for: Two Adjacent cis-Regulatory Elements Are Required for Ecdysone Response of Ecdysone Receptor (EcR) B1 Transcription
Source: PLoS One. 2012 Nov 14;7(11):e49348. doi: 10.1371/journal.pone.0049348 (PMC3498158; doi:10.1371/journal.pone.0049348)
Supplement: Table S5 — List of Primer. (PPT) [file pone.0049348.s012.ppt]

## Slide 1
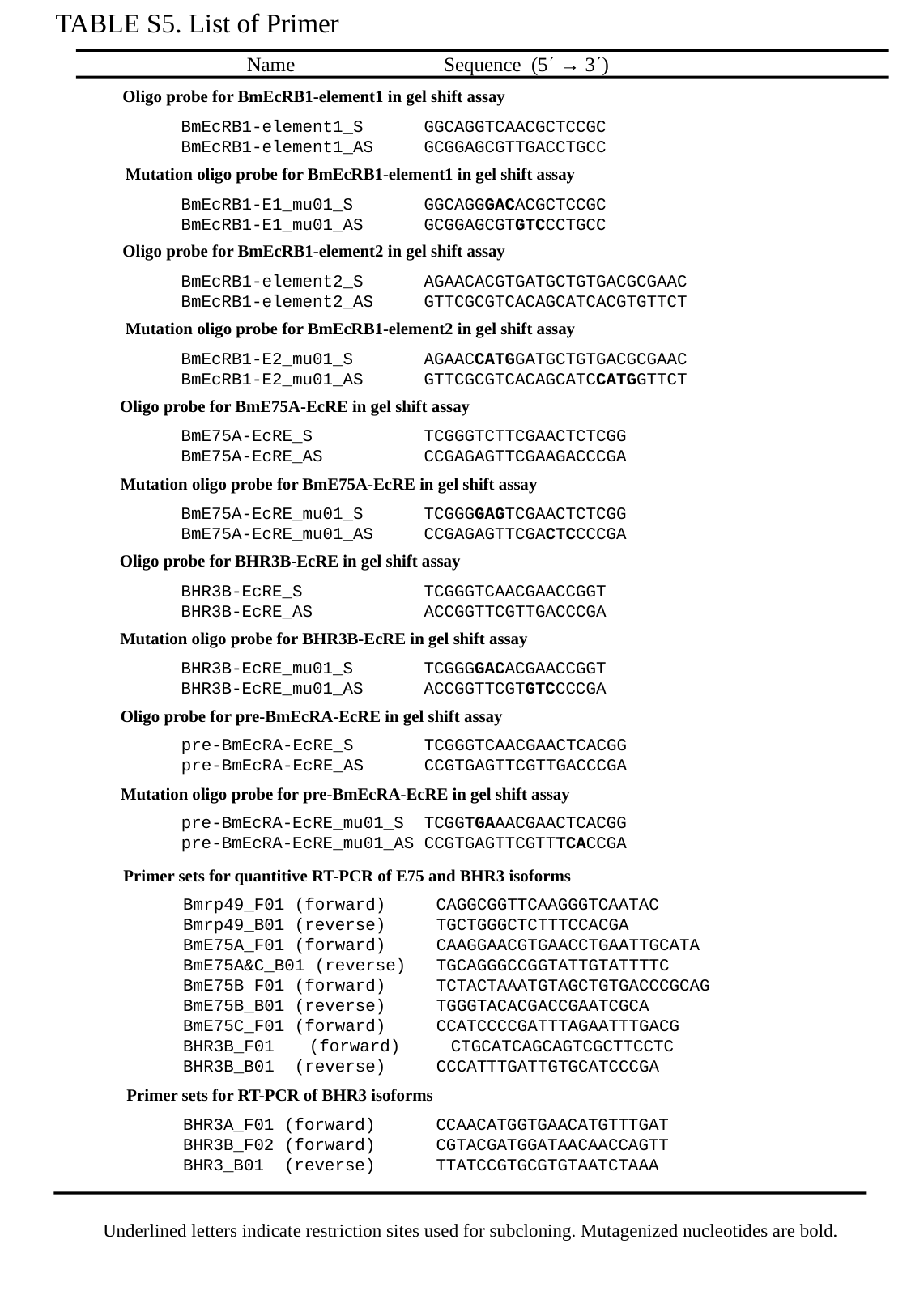

TABLE S5. List of Primer
Name
Sequence (5′ → 3′)
Oligo probe for BmEcRB1-element1 in gel shift assay
BmEcRB1-element1_S	GGCAGGTCAACGCTCCGC
BmEcRB1-element1_AS 	GCGGAGCGTTGACCTGCC
Mutation oligo probe for BmEcRB1-element1 in gel shift assay
BmEcRB1-E1_mu01_S	GGCAGGGACACGCTCCGC
BmEcRB1-E1_mu01_AS	GCGGAGCGTGTCCCTGCC
Oligo probe for BmEcRB1-element2 in gel shift assay
BmEcRB1-element2_S	AGAACACGTGATGCTGTGACGCGAAC
BmEcRB1-element2_AS 	GTTCGCGTCACAGCATCACGTGTTCT
Mutation oligo probe for BmEcRB1-element2 in gel shift assay
BmEcRB1-E2_mu01_S 	AGAACCATGGATGCTGTGACGCGAAC
BmEcRB1-E2_mu01_AS 	GTTCGCGTCACAGCATCCATGGTTCT
Oligo probe for BmE75A-EcRE in gel shift assay
BmE75A-EcRE_S	TCGGGTCTTCGAACTCTCGG
BmE75A-EcRE_AS 	CCGAGAGTTCGAAGACCCGA
Mutation oligo probe for BmE75A-EcRE in gel shift assay
BmE75A-EcRE_mu01_S 	TCGGGGAGTCGAACTCTCGG
BmE75A-EcRE_mu01_AS 	CCGAGAGTTCGACTCCCCGA
Oligo probe for BHR3B-EcRE in gel shift assay
BHR3B-EcRE_S 	TCGGGTCAACGAACCGGT
BHR3B-EcRE_AS 	ACCGGTTCGTTGACCCGA
Mutation oligo probe for BHR3B-EcRE in gel shift assay
BHR3B-EcRE_mu01_S 	TCGGGGACACGAACCGGT
BHR3B-EcRE_mu01_AS 	ACCGGTTCGTGTCCCCGA
Oligo probe for pre-BmEcRA-EcRE in gel shift assay
pre-BmEcRA-EcRE_S 	TCGGGTCAACGAACTCACGG
pre-BmEcRA-EcRE_AS 	CCGTGAGTTCGTTGACCCGA
Mutation oligo probe for pre-BmEcRA-EcRE in gel shift assay
pre-BmEcRA-EcRE_mu01_S 	TCGGTGAAACGAACTCACGG
pre-BmEcRA-EcRE_mu01_AS CCGTGAGTTCGTTTCACCGA
Primer sets for quantitive RT-PCR of E75 and BHR3 isoforms
Bmrp49_F01 (forward) 	 CAGGCGGTTCAAGGGTCAATAC
Bmrp49_B01 (reverse) TGCTGGGCTCTTTCCACGA
BmE75A_F01 (forward) CAAGGAACGTGAACCTGAATTGCATA
BmE75A&C_B01 (reverse) TGCAGGGCCGGTATTGTATTTTC
BmE75B F01 (forward) TCTACTAAATGTAGCTGTGACCCGCAG
BmE75B_B01 (reverse) TGGGTACACGACCGAATCGCA
BmE75C_F01 (forward) CCATCCCCGATTTAGAATTTGACG
BHR3B_F01　 (forward) CTGCATCAGCAGTCGCTTCCTC
BHR3B_B01 (reverse) CCCATTTGATTGTGCATCCCGA
Primer sets for RT-PCR of BHR3 isoforms
BHR3A_F01 (forward) 	 CCAACATGGTGAACATGTTTGAT
BHR3B_F02 (forward) CGTACGATGGATAACAACCAGTT
BHR3_B01 (reverse) TTATCCGTGCGTGTAATCTAAA
Underlined letters indicate restriction sites used for subcloning. Mutagenized nucleotides are bold.
