# Supplementary material for: Two Adjacent cis-Regulatory Elements Are Required for Ecdysone Response of Ecdysone Receptor (EcR) B1 Transcription
Source: PLoS One. 2012 Nov 14;7(11):e49348. doi: 10.1371/journal.pone.0049348 (PMC3498158; doi:10.1371/journal.pone.0049348)
Supplement: Table S6 — List of 20E-inducible genes identified by microarray analysis and the 14 bp consensus motifs. (PPT) [file pone.0049348.s013.ppt]

## Slide 1
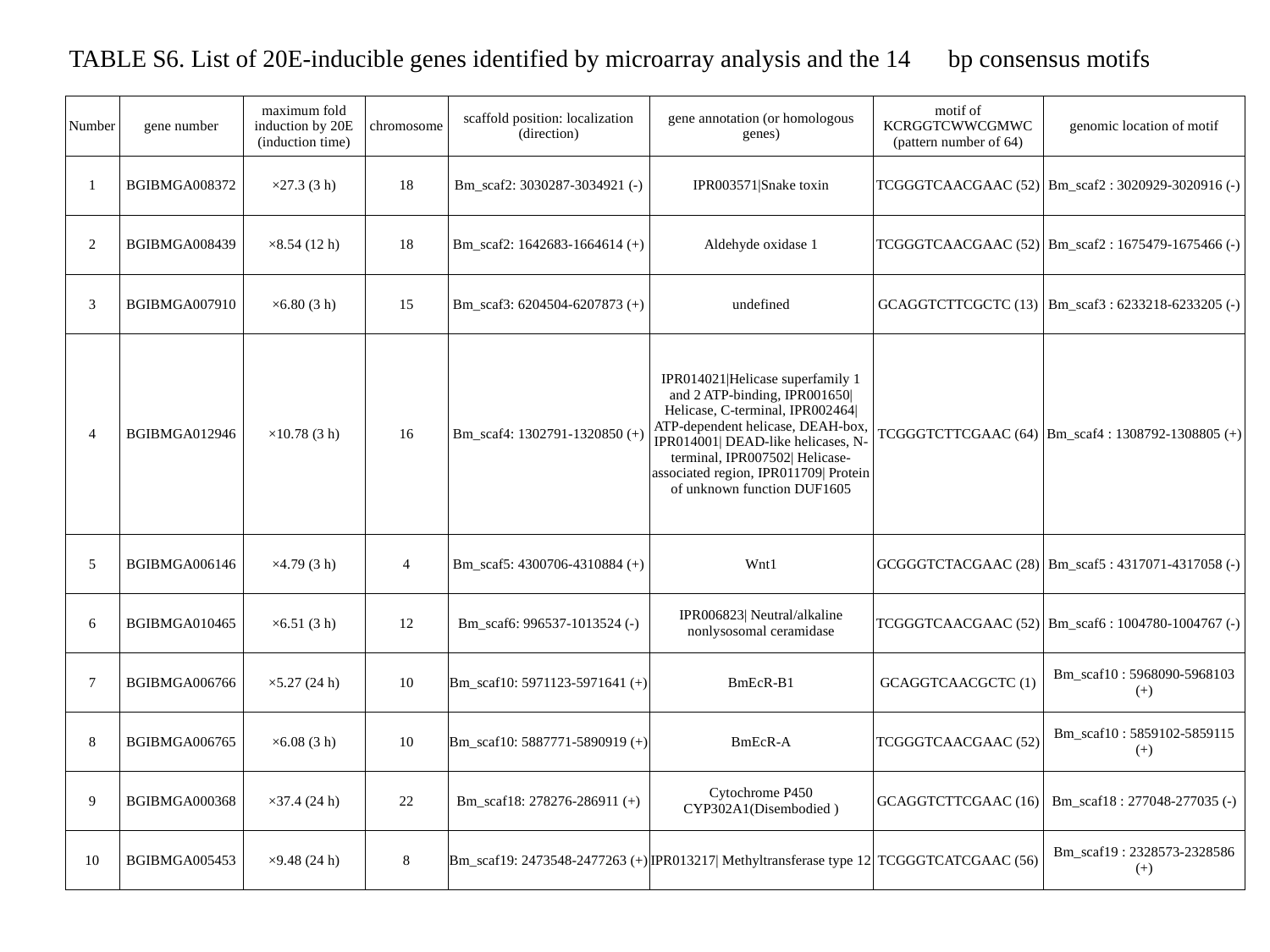

TABLE S6. List of 20E-inducible genes identified by microarray analysis and the 14　bp consensus motifs
| Number | gene number | maximum fold induction by 20E (induction time) | chromosome | scaffold position: localization (direction) | gene annotation (or homologous genes) | motif of KCRGGTCWWCGMWC (pattern number of 64) | genomic location of motif |
| --- | --- | --- | --- | --- | --- | --- | --- |
| 1 | BGIBMGA008372 | ×27.3 (3 h) | 18 | Bm\_scaf2: 3030287-3034921 (-) | IPR003571|Snake toxin | TCGGGTCAACGAAC (52) | Bm\_scaf2 : 3020929-3020916 (-) |
| 2 | BGIBMGA008439 | ×8.54 (12 h) | 18 | Bm\_scaf2: 1642683-1664614 (+) | Aldehyde oxidase 1 | TCGGGTCAACGAAC (52) | Bm\_scaf2 : 1675479-1675466 (-) |
| 3 | BGIBMGA007910 | ×6.80 (3 h) | 15 | Bm\_scaf3: 6204504-6207873 (+) | undefined | GCAGGTCTTCGCTC (13) | Bm\_scaf3 : 6233218-6233205 (-) |
| 4 | BGIBMGA012946 | ×10.78 (3 h) | 16 | Bm\_scaf4: 1302791-1320850 (+) | IPR014021|Helicase superfamily 1 and 2 ATP-binding, IPR001650|Helicase, C-terminal, IPR002464|ATP-dependent helicase, DEAH-box, IPR014001| DEAD-like helicases, N-terminal, IPR007502| Helicase-associated region, IPR011709| Protein of unknown function DUF1605 | TCGGGTCTTCGAAC (64) | Bm\_scaf4 : 1308792-1308805 (+) |
| 5 | BGIBMGA006146 | ×4.79 (3 h) | 4 | Bm\_scaf5: 4300706-4310884 (+) | Wnt1 | GCGGGTCTACGAAC (28) | Bm\_scaf5 : 4317071-4317058 (-) |
| 6 | BGIBMGA010465 | ×6.51 (3 h) | 12 | Bm\_scaf6: 996537-1013524 (-) | IPR006823| Neutral/alkaline nonlysosomal ceramidase | TCGGGTCAACGAAC (52) | Bm\_scaf6 : 1004780-1004767 (-) |
| 7 | BGIBMGA006766 | ×5.27 (24 h) | 10 | Bm\_scaf10: 5971123-5971641 (+) | BmEcR-B1 | GCAGGTCAACGCTC (1) | Bm\_scaf10 : 5968090-5968103 (+) |
| 8 | BGIBMGA006765 | ×6.08 (3 h) | 10 | Bm\_scaf10: 5887771-5890919 (+) | BmEcR-A | TCGGGTCAACGAAC (52) | Bm\_scaf10 : 5859102-5859115 (+) |
| 9 | BGIBMGA000368 | ×37.4 (24 h) | 22 | Bm\_scaf18: 278276-286911 (+) | Cytochrome P450 CYP302A1(Disembodied ) | GCAGGTCTTCGAAC (16) | Bm\_scaf18 : 277048-277035 (-) |
| 10 | BGIBMGA005453 | ×9.48 (24 h) | 8 | Bm\_scaf19: 2473548-2477263 (+) | IPR013217| Methyltransferase type 12 | TCGGGTCATCGAAC (56) | Bm\_scaf19 : 2328573-2328586 (+) |

## Slide 2
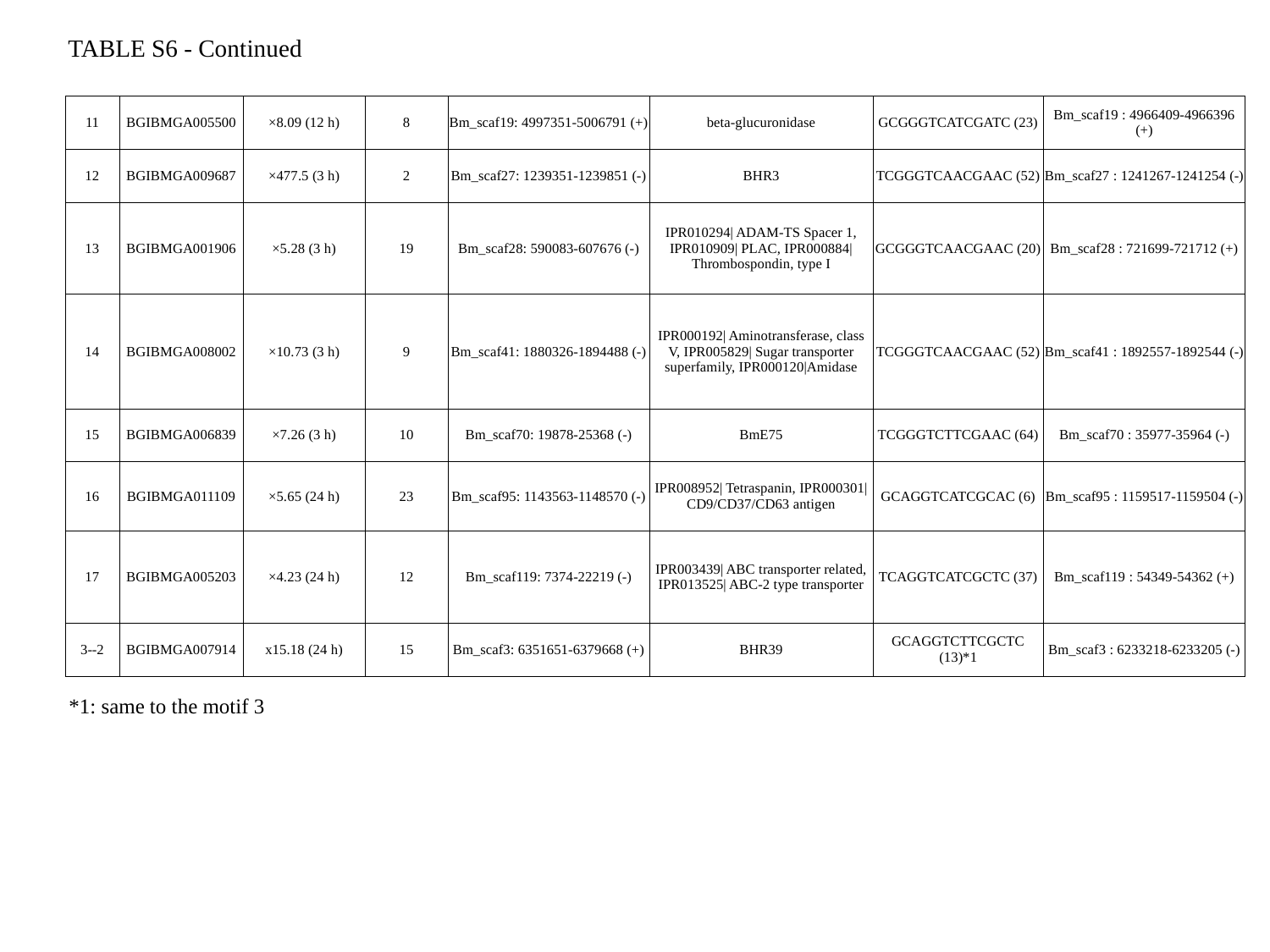

TABLE S6 - Continued
| 11 | BGIBMGA005500 | ×8.09 (12 h) | 8 | Bm\_scaf19: 4997351-5006791 (+) | beta-glucuronidase | GCGGGTCATCGATC (23) | Bm\_scaf19 : 4966409-4966396 (+) |
| --- | --- | --- | --- | --- | --- | --- | --- |
| 12 | BGIBMGA009687 | ×477.5 (3 h) | 2 | Bm\_scaf27: 1239351-1239851 (-) | BHR3 | TCGGGTCAACGAAC (52) | Bm\_scaf27 : 1241267-1241254 (-) |
| 13 | BGIBMGA001906 | ×5.28 (3 h) | 19 | Bm\_scaf28: 590083-607676 (-) | IPR010294| ADAM-TS Spacer 1, IPR010909| PLAC, IPR000884| Thrombospondin, type I | GCGGGTCAACGAAC (20) | Bm\_scaf28 : 721699-721712 (+) |
| 14 | BGIBMGA008002 | ×10.73 (3 h) | 9 | Bm\_scaf41: 1880326-1894488 (-) | IPR000192| Aminotransferase, class V, IPR005829| Sugar transporter superfamily, IPR000120|Amidase | TCGGGTCAACGAAC (52) | Bm\_scaf41 : 1892557-1892544 (-) |
| 15 | BGIBMGA006839 | ×7.26 (3 h) | 10 | Bm\_scaf70: 19878-25368 (-) | BmE75 | TCGGGTCTTCGAAC (64) | Bm\_scaf70 : 35977-35964 (-) |
| 16 | BGIBMGA011109 | ×5.65 (24 h) | 23 | Bm\_scaf95: 1143563-1148570 (-) | IPR008952| Tetraspanin, IPR000301| CD9/CD37/CD63 antigen | GCAGGTCATCGCAC (6) | Bm\_scaf95 : 1159517-1159504 (-) |
| 17 | BGIBMGA005203 | ×4.23 (24 h) | 12 | Bm\_scaf119: 7374-22219 (-) | IPR003439| ABC transporter related, IPR013525| ABC-2 type transporter | TCAGGTCATCGCTC (37) | Bm\_scaf119 : 54349-54362 (+) |
| 3--2 | BGIBMGA007914 | x15.18 (24 h) | 15 | Bm\_scaf3: 6351651-6379668 (+) | BHR39 | GCAGGTCTTCGCTC (13)\*1 | Bm\_scaf3 : 6233218-6233205 (-) |
*1: same to the motif 3
